# Supplementary material for: Patient-level and practice-level factors associated with consultation duration: a cross-sectional analysis of over one million consultations in English primary care
Source: BMJ Open. 2017 Nov 16;7(11):e018261. doi: 10.1136/bmjopen-2017-018261 (PMC5701995; doi:10.1136/bmjopen-2017-018261)
Supplement: Supplementary file 1 [file bmjopen-2017-018261supp001.pdf]

Supplement

Figure S1: Distribution of mean consultation duration in each practice for face-to-face or telephone consultations conducted by a GP or nurse

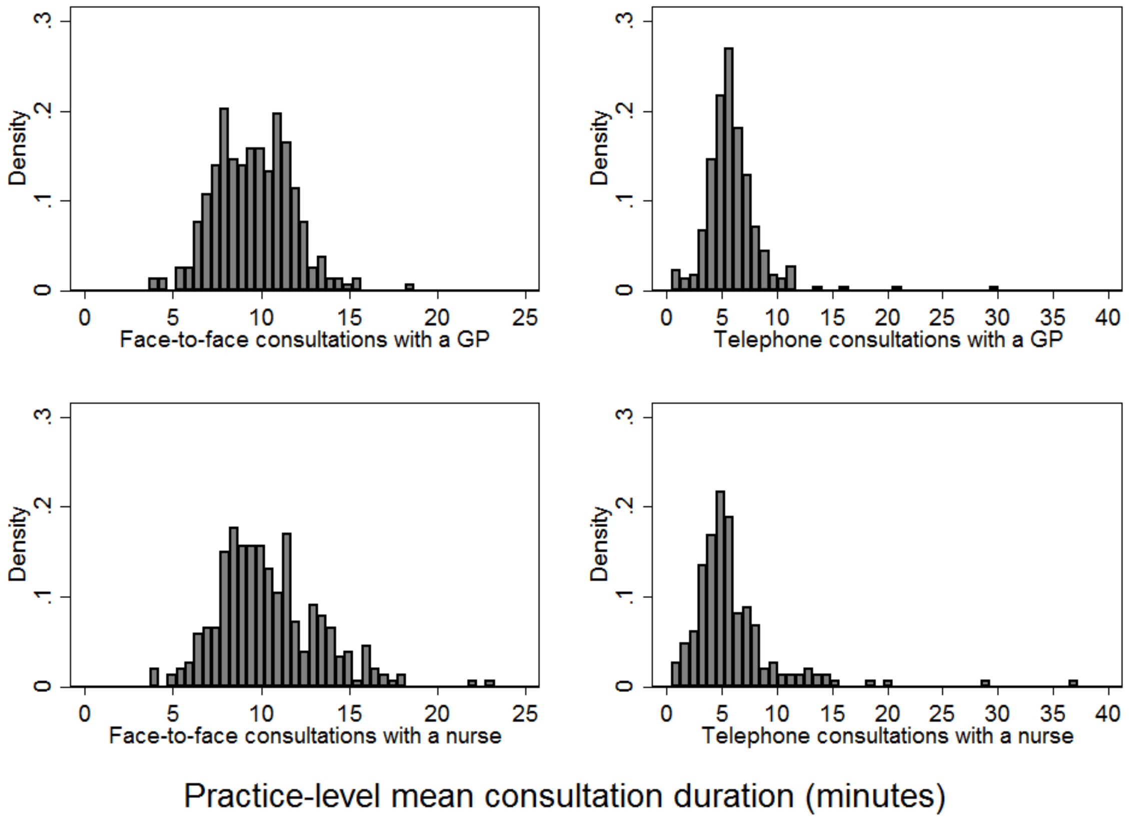

Table S1: Characteristics of practices according to mean duration of consultation with a GP

	Practice mean consultation duration (minutes) (N=316)					
	**<5**	**≥5 and <8**	**≥8 and <10**	**≥10 and <12**	**≥12 and <15**	**≥15**
**Number of practices (%)**	**4 (1.3)**	**96 (30.4)**	**118 (37.3)**	**83 (26.3)**	**14 (4.4)**	**1 (0.3)**
Mean list size (SD)	5,580.0 (713.0)	10,149.3 (4,603.6)	10,232.1 (4809.5)	8,771.6 (4,590.9)	7,715.2 (2,912.3)	9,220.0 (-)
Training practice						
Yes: N (%)	0 (0.0)	33 (34.4)	53 (44.9)	35 (42.2)	5 (35.7)	0 (0.0)
Rurality						
Not rural (Urban >10K - less sparse):N (%)	4 (100.0)	82 (85.4)	101 (85.6)	68 (81.9)	12 (85.7)	0 (0.0)
Mean GP consultation rate (per 10,000 person years) (SD)	46,877.5 (17,478.6)	42,488.2 (16,711.8)	37,671.8 (9,954.9)	32,618.1 (9,227.8)	28,463.9 (9,387.5)	13,895.5 (-)
Mean nurse consultation rate (per 10,000 person years) (SD)	12,777.7 (4,657.8)	14,270.9 (7,893.9)	13,566.1 (7,677.1)	11,707.4 (6,888.7)	12,279.2 (8,778.4)	11,124.7 (-)
Number of FTE GPs						
<=2: N (%)	2 (50.0)	9 (9.4)	16 (13.6)	14 (16.9)	3 (21.4)	0 (0.0)
>2 and <=4: N (%)	2 (50.0)	24 (25.0)	22 (18.6)	24 (28.9)	2 (14.3)	0 (0.0)
>4 and <=6: N (%)	0 (0.0)	37 (38.5)	32 (27.1)	24 (28.9)	7 (50.0)	1 (100.0)
>6 and <=8: N (%)	0 (0.0)	15 (15.6)	25 (21.2)	15 (18.1)	0 (0.0)	0 (0.0)
>8 and <=19: N (%)	0 (0.0)	11 (11.5)	22 (18.6)	5 (6.0)	2(14.3)	0 (0.0)
Number of FTE nurses						
<=2: N (%)	4 (100.0)	51 (53.1)	69 (58.5)	52 (62.7)	12 (85.7)	0 (0.0)
>2 and <=4: N (%)	0 (0.0)	22 (22.9)	22 (18.6)	18 (21.7)	2 (14.3)	1 (100.0)
>4 and <=6: N (%)	0 (0.0)	8 (8.3)	10 (8.5)	2 (2.4)	0 (0.0)	0 (0.0)
>6 and <=8: N (%)	0 (0.0)	4 (4.2)	2 (1.7)	0 (0.0)	0 (0.0)	0 (0.0)
>8 and <=19: N (%)	0 (0.0)	2 (2.1)	2 (1.7)	0 (0.0)	0 (0.0)	0 (0.0)
QOF performance						
1st quintile (poorest performance): N (%)	2 (50.0)	15 (15.6)	18 (15.3)	12 (14.5)	3 (21.4)	0 (0.0)
2nd quintile: N (%)	0 (0.0)	22(22.9)	13 (11.0)	13 (15.7)	1 (7.1)	0 (0.0)
3rd quintile: N (%)	1 (25.0)	23 (24.0)	24 (20.3)	8 (9.6)	2 (14.3)	1 (100.0)
4th quintile: N (%)	1 (25.0)	18 (18.8)	31 (26.3)	27 (32.5)	5 (35.7)	0 (0.0)
5th quintile (best performance): N (%)	0 (0.0)	18 (18.8)	30 (25.4)	22 (26.5)	3 (21.4)	0 (0.0)

Table S2: Full model for consultations with a GP

	Change in duration (seconds)	p-value	95% confidence interval	
Female gender (Male = reference)	8.29	0.000	6.03	10.55
Ethnic group (White = reference)				
Asian	4.11	0.231	-2.61	10.84
Chinese	-6.32	0.607	-30.41	17.76
Black	-5.63	0.205	-14.34	3.07
Mixed/ Other	4.35	0.283	-3.59	12.29
Unknown	-11.00	0.000	-13.50	-8.49
Index of multiple deprivation (1st quintile = reference)				
2nd quintile	1.14	0.535	-2.47	4.76
3rd quintile	-2.39	0.235	-6.33	1.55
4th quintile	-3.57	0.095	-7.77	0.62
5th quintile (most deprived)	-5.04	0.037	-9.77	-0.30
Unknown	-11.27	0.060	-23.02	0.48
Smoking status (Non-smoker = reference)				
Current smoker	-2.36	0.147	-5.54	0.83
Ex-smoker	0.11	0.944	-2.91	3.13
Unknown	18.66	0.000	14.58	22.75
Age group (0-14 years = reference)				
15-24	55.71	0.000	50.12	61.30
25-44	83.69	0.000	78.72	88.65
45-64	89.83	0.000	84.77	94.89
65-74	65.84	0.000	60.25	71.42
75+	58.44	0.000	52.96	63.93
Telephone consultation (Face-to-face = reference)	-308.70	0.000	-311.64	-305.76
Practice list size (centred, per 1000)	-5.21	0.026	-9.81	-0.61
Number of FTE GPs (≤2 = reference)				
>2 and <=4	27.74	0.206	-15.23	70.70
>4 and <=6	56.90	0.016	10.62	103.18
>6 and <=8	58.06	0.061	-2.61	118.73
>8 and <=19	120.02	0.002	43.58	196.47
Unknown	211.75	0.120	-54.93	478.44
Number of FTE nurses (≤2 = reference)				
>2 and <=4	-10.07	0.568	-44.59	24.46
>4 and <=6	-36.07	0.239	-96.11	23.97
>6 and <=8	-141.57	0.003	-235.44	-47.70
>8 and <=19	54.83	0.354	-61.14	170.81
Unknown	-16.52	0.429	-57.41	24.37
Training practice (No = reference)				
Yes	45.61	0.001	18.26	72.96
Unknown	-			
Nurse consultation rate (centred, per 1000 per 10,000 person years)	0.40	0.658	-1.36	2.15
GP consultation rate (centred, per 1000 per 10,000 person years)	-3.83	0.000	-4.80	-2.85
QOF performance (1st quintile = reference)				
2nd quintile	3.50	0.869	-38.00	44.99
3rd quintile	-16.22	0.427	-56.19	23.76
4th quintile	27.47	0.147	-9.67	64.61
5th quintile (best performance)	18.21	0.343	-19.40	55.83
Unknown	-50.43	0.633	-257.64	156.78
Rural practice (Urban = reference)	17.21	0.309	-15.98	50.39
Mean duration	417.27	0.000	367.50	467.04

Table S3: Final model for consultations with a GP including a term for GP registrar role (post-hoc sensitivity analysis)

	Change in duration (seconds)	p-value	95% confidence interval	
Female gender (Male = reference)	7.40	0.000	5.16	9.63
Ethnic group (White = reference)				
Asian	4.54	0.181	-2.11	11.19
Chinese	-7.76	0.523	-31.58	16.07
Black	-7.18	0.102	-15.79	1.43
Mixed/ Other	3.82	0.340	-4.03	11.68
Unknown	-12.32	0.000	-14.80	-9.85
Index of multiple deprivation (1st quintile = reference)				
2nd quintile	0.52	0.776	-3.06	4.10
3rd quintile	-3.37	0.090	-7.27	0.53
4th quintile	-4.88	0.021	-9.03	-0.74
5th quintile (most deprived)	-6.62	0.006	-11.30	-1.94
Unknown	-8.24	0.165	-19.87	3.39
Smoking status (Non-smoker = reference)				
Current smoker	-3.11	0.053	-6.26	0.04
Ex-smoker	-0.10	0.945	-3.09	2.88
Unknown	15.56	0.000	11.52	19.60
Age group (0-14 years = reference)				
15-24	52.35	0.000	46.82	57.88
25-44	81.88	0.000	76.97	86.79
45-64	92.15	0.000	87.14	97.15
65-74	71.73	0.000	66.21	77.26
75+	64.95	0.000	59.52	70.38
Telephone consultation (Face-to-face = reference)	-295.45	0.000	-298.37	-292.53
Training practice (No = reference)				
Yes	21.92	0.073	-2.07	45.90
Unknown	133.11	0.107	-28.55	294.77
GP registrar conducting appointment (No=reference)	245.04	0.000	241.49	248.59
GP consultation rate (centred, per 1000 per 10,000 person years)	-3.33	0.000	-4.24	-2.41
Mean duration	460.60	0.000	443.91	477.30

Table S4: Characteristics of practices according to mean duration of consultation with a nurse

	Practice mean consultation duration (minutes) (N=307)					
	**<5**	**≥5 and <8**	**≥8 and <10**	**≥10 and <12**	**≥12 and <15**	**≥15**
**Number of practices (%)**	**3 (1.0)**	**59 (19.2)**	**103 (33.6)**	**71 (23.1)**	**54 (17.6)**	**17 (5.5)**
Mean list size (SD)	8,189.0 (6,263.3)	9,936.8 (4,101.1)	10,029.6 (4,506.8)	10,945.5 (5,387.0)	8,591.0 (3,987.7)	7,286.6 (3,564.8)
Training practice						
Yes: N (%)	2 (66.7)	24 (40.7)	43 (41.8)	37 (52.1)	15 (27.8)	5 (29.4)
Rurality						
Not rural (Urban >10K - less sparse):N (%)	3 (100.0)	50 (84.8)	86 (83.5)	59 (83.1)	47 (87.0)	15 (88.2)
Mean GP consultation rate (per 10,000 person years) (SD)	34,558.4 (7,497.0)	39,556.1 (17,065.8)	39,333.7 (13,086.5)	37,073.1 (11,166.8)	34,315.5 (8,097.0)	35,907.5 (13,077.5)
Mean nurse consultation rate (per 10,000 person years) (SD)	15,359.3 (18,373.0)	17,422.4 (7,482.5)	15,404.9 (7,757.8)	12,162.1 (5,803.8)	9,868.6 (4,282.7)	7,029.6 (3,980.1)
Number of FTE GPs						
<=2: N (%)	1 (33.3)	5 (8.5)	10 (9.7)	6 (8.5)	12 (22.2)	5 (29.4)
>2 and <=4: N (%)	1 (33.3)	14 (23.7)	28 (27.2)	10 (14.1)	16 (29.6)	4 (23.5)
>4 and <=6: N (%)	0 (0.0)	22 (37.3)	27 (26.2)	31 (43.4)	13 (24.1)	6 (35.3)
>6 and <=8: N (%)	1 (33.3)	10 (17.0)	22 (21.4)	11 (15.5)	9 (16.7)	2 (11.8)
>8 and <=19: N (%)	0 (0.0)	7 (11.9)	16 (15.5)	13 (18.3)	4 (7.4)	0 (0.0)
Number of FTE nurses						
<=2: N (%)	2 (66.7)	35 (59.3)	51 (49.5)	42 (59.2)	38 (70.4)	12 (70.6)
>2 and <=4: N (%)	1 (33.3)	12 (20.3)	28 (27.2)	15 (21.2)	7 (13.0)	2 (11.8)
>4 and <=6: N (%)	0 (0.0)	3 (5.1)	11 (10.7)	5 (7.0)	1 (19.9)	0 (0.0)
>6 and <=8: N (%)	0 (0.0)	1 (1.7)	0 (0.0)	4 (5.6)	1 (1.9)	0 (0.0)
>8 and <=19: N (%)	0 (0.0)	1 (1.7)	2 (1.9)	1 (1.4)	0 (0.0)	0 (0.0)
QOF performance						
1st quintile (poorest performance): N (%)	1 (33.3)	9 (15.3)	15 (14.6)	13 (18.3)	7 (13.0)	2 (11.8)
2nd quintile: N (%)	0 (0.0)	5 (8.5)	17 (16.5)	13 (18.3)	10 (18.5)	1 (5.9)
3rd quintile: N (%)	0 (0.0)	9 (15.3)	24 (23.3)	13 (18.3)	7 (13.0)	6 (35.3)
4th quintile: N (%)	1 (33.3)	17 (28.8)	28 (27.2)	20 (28.2)	11 (20.4)	4 (23.5)
5th quintile (best performance): N (%)	1 (33.3)	17 (28.8)	19 (18.5)	12 (16.9)	19 (35.2)	4 (23.5)

Table S5: Full model for consultations with a nurse

	Change in duration (seconds)	p-value	95% confidence interval	
Female gender (Male = reference)	-11.15	0.000	-15.34	-6.96
Ethnic group (White = reference)				
Asian	0.18	0.979	-12.99	13.35
Chinese	-17.82	0.445	-63.57	27.93
Black	-1.67	0.849	-18.84	15.50
Mixed/ Other	-5.52	0.489	-21.18	10.13
Unknown	-2.52	0.279	-7.09	2.04
Index of multiple deprivation (1st quintile = reference)				
2nd quintile	7.49	0.026	0.92	14.06
3rd quintile	-0.54	0.883	-7.70	6.62
4th quintile	5.38	0.167	-2.24	13.01
5th quintile (most deprived)	7.92	0.071	-0.67	16.51
Unknown	-25.47	0.011	-45.10	-5.84
Smoking status (Non-smoker = reference)				
Current smoker	26.40	0.000	20.49	32.31
Ex-smoker	15.00	0.000	9.59	20.42
Unknown	21.12	0.000	13.23	29.01
Age group (0-14 years = reference)				
15-24	52.88	0.000	42.16	63.60
25-44	72.19	0.000	62.83	81.55
45-64	113.49	0.000	103.97	123.00
65-74	73.96	0.000	63.81	84.10
75+	75.54	0.000	65.43	85.65
Telephone consultation (Face-to-face = reference)	-279.51	0.000	-288.35	-270.66
Practice list size (centred, per 1000)	-6.09	0.065	-12.56	0.38
Number of FTE GPs (≤2 = reference)				
>2 and <=4	-63.99	0.045	-126.58	-1.39
>4 and <=6	-49.06	0.150	-115.82	17.70
>6 and <=8	-33.73	0.442	-119.68	52.22
>8 and <=19	-29.10	0.596	-136.61	78.42
Unknown	-106.02	0.625	-531.17	319.12
Number of FTE nurses (≤2 = reference)				
>2 and <=4	20.31	0.408	-27.79	68.41
>4 and <=6	49.99	0.240	-33.47	133.46
>6 and <=8	113.35	0.088	-16.77	243.48
>8 and <=19	137.25	0.096	-24.40	298.89
Unknown	-7.81	0.791	-65.58	49.97
Training practice (No = reference)				
Yes	-11.09	0.570	-49.31	27.14
Unknown	-			
Nurse consultation rate, centred, per 1000 per 10,000 person years)	-10.23	0.000	-12.84	-7.62
GP consultation rate, centred, per 1000 per 10,000 person years)	-0.49	0.488	-1.88	0.90
QOF performance (1st quintile = reference)				
2nd quintile	28.82	0.345	-30.93	88.56
3rd quintile	6.08	0.835	-50.99	63.14
4th quintile	9.11	0.738	-44.24	62.47
5th quintile (best performance)	31.22	0.254	-22.44	84.89
Unknown	-81.78	0.575	-367.98	204.42
Rural practice (Urban = reference)	13.93	0.561	-33.09	60.96
Mean duration	543.41	0.000	470.92	615.90
