# Supplementary material for: Patient-level and practice-level factors associated with consultation duration: a cross-sectional analysis of over one million consultations in English primary care
Source: BMJ Open. 2017 Nov 16;7(11):e018261. doi: 10.1136/bmjopen-2017-018261 (PMC5701995; doi:10.1136/bmjopen-2017-018261)
Supplement: Author's manuscript [file bmjopen-2017-018261.draft_revisions.pdf]

BMJ Open

BMJ Open is committed to open peer review. As part of this commitment we make the peer review history of every article we publish publicly available.

When an article is published we post the peer reviewers' comments and the authors' responses online. We also post the versions of the paper that were used during peer review. These are the versions that the peer review comments apply to.

The versions of the paper that follow are the versions that were submitted during the peer review process. They are not the versions of record or the final published versions. They should not be cited or distributed as the published version of this manuscript.

BMJ Open is an open access journal and the full, final, typeset and author-corrected version of record of the manuscript is available on our site with no access controls, subscription charges or pay-per-view fees (http://bmjopen.bmj.com).

If you have any questions on BMJ Open's open peer review process please email editorial.bmjopen@bmj.com

BMJ Open

Patient and practice level factors associated with consultation duration: a cross-sectional analysis of over 1 million consultations in English primary care.

Journal:	*BMJ Open*
Manuscript ID	bmjopen-2017-018261
Article Type:	Research
Date Submitted by the Author:	15-Jun-2017
Complete List of Authors:	Stevens, Sarah; University of Oxford, Nuffield Department of Primary Care Health Sciences<br>Bankhead, Clare; University of Oxford<br>Mukhtar, Toqir; University of Oxford<br>Perera, Rafael; University of Oxford, Primary Health Care<br>Holt, Tim; Oxford University, Department of Primary Care Health Sciences<br>Salisbury, Chris; University of Bristol, Academic Unit of Primary Health Care<br>Hobbs, Richard; University of Oxford, Dept of Primary Health Care
**Primary Subject Heading**:	General practice / Family practice
Secondary Subject Heading:	Health services research
Keywords:	PRIMARY CARE, Quality in health care < HEALTH SERVICES ADMINISTRATION & MANAGEMENT, Organisation of health services < HEALTH SERVICES ADMINISTRATION & MANAGEMENT, Health policy < HEALTH SERVICES ADMINISTRATION & MANAGEMENT

SCHOLARONE™
Manuscripts

For peer review only

Patient and practice level factors associated with consultation duration: a cross-sectional analysis of over 1 million consultations in English primary care.

Sarah Stevens[1], Clare Bankhead[1], Toqir Mukhtar[1], Rafael Perera-Salazar[1], Tim Holt[1], Chris Salisbury[2], FD Richard Hobbs[1], on behalf of the NIHR School for Primary Care Research, Nuffield Department of Primary Care Health Sciences, University of Oxford

1. Nuffield Department of Primary Care Health Sciences, University of Oxford, Oxford, UK
2. Centre for Academic Primary Care, School of Social and Community Medicine, University of Bristol, Bristol, UK.

Corresponding author:

Sarah Stevens,

Contact details: sarah.stevens@phc.ox.ac.uk, 01865289449

Address: Nuffield Department of Primary Care Health Sciences, Radcliffe Primary Care Building, Radcliffe Observatory Quarter, Woodstock Road, Oxford, OX2 6GG;

Word count: 3000

For peer review only

ABSTRACT

Objectives: Consultation duration has previously been shown to be associated with patient, practitioner, and practice level characteristics. However, previous studies were conducted in countries other than the UK, considered only small numbers of GP consultations, or focused primarily on practitioner level characteristics. We aimed to determine the patient and practice level factors associated with duration of GP and nurse consultations in UK primary care.

Design and setting: Cross sectional data were obtained from English general practices contributing to the Clinical Practice Research Datalink (CPRD) linked to data on patient deprivation and practice staffing, rurality, and Quality and Outcomes Framework (QOF) achievement.

Participants: 218,304 patients, from 316 English general practices, consulting from 1st April 2013 to 31st March 2014.

Analysis: Multilevel mixed effects models described the association between consultation duration and patient and practice-level factors (patient age, gender, smoking status, ethnic group, deprivation and practice rurality, number of full time equivalent GPs/nurses, list size, consultation rate, quintile of overall QOF achievement, and training status).

Results: Mean duration of face-to-face GP consultations was 9.24 minutes compared to 5.32 minutes for telephone consultations. Nurse face-to-face and telephone nurse consultations lasted 9.70 and 5.73 minutes on average, respectively. In 964,148 GP consultations, duration was associated with patient gender, age, deprivation, and practice training status, and practice consultation rate. In 347,657 nurse consultations, duration was associated with patient gender, age, smoking status, and practice consultation rate. Observed differences in duration were small (e.g. GP consultations with female patients were 8 seconds longer than those with male patients on average).

Conclusions: Small observed differences in consultation duration indicate that patients are treated similarly regardless of background. Increased consultation duration may be beneficial for older or comorbid patients, but the benefits and costs of increased consultation duration require further study.

ARTICLE SUMMARY

Strengths and limitations of this study

- This is a large-scale analysis of over 1 million consultations, using data known to representative of the UK population.
- We have considered factors associated with the duration of both GP and nurse consultations allowing comparison between the two.
- Appointment duration may be recorded with some error, but average durations were consistent with 10-minute appointment slots.
- We were unable to examine how GP / nurse characteristics are associated with consultation duration and this requires further study.

For peer review only

INTRODUCTION

Patient-facing general practice workload in England has increased by 16% since 2007.[1] This reflects an increase in both the consultation rate and the duration of consultations. Consultation duration may be influenced by patient, practitioner, and practice level characteristics. At the practice level, previous studies have shown that shorter consultation duration is associated with greater practice list size[2] and greater practice workload.[3] The influence of practice rurality (rural compared to urban) is unclear with some studies indicating that rurality is negatively associated with consultation duration[2,4], and others demonstrating a positive association.[5] Relevant practitioner characteristics associated with longer consultations include female gender,[6] older age[3,5], but conversely, lesser experience.[6] Finally, longer consultations have been shown to be associated with patient characteristics, including female gender,[3–5,7] greater age,[2–5,7] greater number of presenting problems,[3–5,7,8] and higher level of education[3] or socioeconomic status.[5]

However, many previous studies have been conducted in countries other than the UK and findings may not be generalizable to the National Health Service.[2–5] Studies within the UK provide limited up-to-date evidence having been conducted some time ago using data on a relatively small number of consultations,[7] or in specific areas, focusing primarily on practitioner level characteristics alone.[6] Although a 2013 paper studied the association between practice, practitioner, and patient level characteristics and the number of presenting problems, demonstrating that the number of presenting problems is also associated with consultation duration, direct links between patient and practice characteristics and duration were not studied.[8] Finally, previous work has considered duration of GP consultations only, despite nurse consultations accounting for approximately one quarter of the overall UK primary care consultation rate in 2013/14.[1] Hence, we aimed to determine the patient and practice level factors associated with increased duration of consultation in UK primary care in contemporary data, considering both GP consultations, and previously unstudied nurse consultations.

METHODS

Consultation and patient level data were obtained from the Clinical Practice Research Datalink (CPRD). CPRD practices in England were eligible for inclusion in the study if they consented to CPRD's data linkage scheme, contributed data covering any part of the study period (1st April 2013 to 31st March 2014), and data were of 'research standard'. Patients were eligible for inclusion if they were non-temporary patients registered at eligible practices for at least one day during the study period. This represents a subset of practices and patients included in a previous study conducted by the authors.[1] Due to the volume of data, this analysis was limited to a 10% random sample from each age-sex strata of eligible patents and those who consulted at least once during the study period.

CPRD data was linked to practice level data on staffing,[9] rurality,[10] and Quality and Outcomes Framework (QOF) performance measures,[11] and to patient level Index of Multiple Deprivation (IMD) and Hospital Episodes Statistics (HES) data. The study group downloaded staffing, rurality, and QOF data from NHS digital (formerly the Health and Social Care Information Centre), and grouped continuous variables before the data was linked to CPRD data. This was a requirement of ethical approval from the Independent Scientific Advisory Committee (ISAC) to CPRD, in order to limit the possibility of identifying individual practices contributing to CPRD. The approved protocol (number 15_120R) is available from the authors on request.

Consultations in CPRD data represent distinct occasions on which a patient's electronic health record is opened by a practice staff member. We analysed consultations that were identified as face-to-face

For peer review only - http://bmjopen.bmj.com/site/about/guidelines.xhtml

or telephone consultations only based on the variable "consultation type", and those with a GP or nurse only, as indicated by the variable "staff role". Hence we excluded consultations representing instances where the patient record was opened purely for administrative purposes by GPs, nurses, or administrative staff (for example to record test results). We also excluded home visit consultations as the recorded duration may merely represent the time taken to record the consultation after it has ended.

Multilevel mixed effects models were used to model the association between patient and practice-level factors and the duration of GP or nurse consultations separately. Patient level factors included as fixed effects were age, gender, smoking status (current, former, never), ethnic group, and deprivation (quintile of IMD). Fixed effects practice level factors included were rurality, number of full time equivalent (FTE) GPs, number of FTE nurses, list size (centred), rate of GP consultation (centred), rate of nurse consultation (centred), quintile of overall QOF achievement, and whether the practice was a training practice (or not). Indicators for the patient and practice were included as random effects. All variables were entered into the models simultaneously and subsequently excluded in a stepwise fashion based on Z tests (binary and continuous variables), or chi-squared tests (categorical variables) at the 5% level. Missing smoking status and ethnic group data was included as a separate category in the models.

RESULTS

In total, 3,049,320 patients were eligible during the study period and 304,937 patients were selected at random for inclusion in this study. Of these, 218,304 consulting patients from 316 practices were included. In these patients during the study period 964,148 consultations were conducted by a GP, and 347,657 were conducted by a nurse (1,311,805 consultations in total). The majority of consultations (1,155,040; 88%) were face-to-face consultations. Face-to-face consultations were, on average, longer than telephone consultations. Mean duration of face-to-face GP consultations was 9.24 (SD=8.06) minutes compared to 5.32 (6.21) minutes for telephone consultations. Nurse consultations were, on average, also slightly longer than those with GPs; face-to-face and telephone nurse consultations lasted 9.70 (9.21) and 5.73 (6.29) minutes. The characteristics of the included patients and practices are given in Table 1 and Table 2, respectively.

For peer review only

For peer review only - http://bmjopen.bmj.com/site/about/guidelines.xhtml

Table 1: Characteristics of included patients (N=218,304)

	Mean/ N	SD/ %
Female gender	121,107	55.5
Age group		
0-14 years	36,371	16.7
15-24	23,020	10.5
25-44	55,316	25.3
45-64	57,000	26.1
65-74	24,086	11.0
75+	22,511	10.3
Smoking status		
Non-smoker	82,327	37.7
Current smoker	37,286	17.1
Ex-smoker	40,834	18.7
Unknown	57,857	26.5
Index of multiple deprivation		
1st quintile (least deprived)	48,363	22.2
2nd quintile	47,948	22.0
3rd quintile	41,825	19.2
4th quintile	41,953	19.2
5th quintile (most deprived)	34,750	15.9
Unknown	3,465	1.6
Ethnic group		
White	118,063	54.1
Asian	6,008	2.8
Chinese	491	0.2
Black	3,908	1.8
Mixed/ Other	4,374	2.0
Unknown	85,460	39.2

For peer review only

For peer review only - http://bmjopen.bmj.com/site/about/guidelines.xhtml

Table 2: Characteristics of included practices (N=316)

	Mean/ N	SD/ %
List size	9,649.7	4648.4
Training practice		
Yes	126	39.9
Unknown	2	0.6
Rurality		
Not rural (Urban >10K - less sparse)	267	84.5
Rural (Hamlet/village/town & fringe)	49	15.5
GP consultation rate (per 10,000 person years)	37,441.0	13,043.6
Nurse consultation rate (per 10,000 person years)	13,217.3	7,580.5
Number of FTE GPs		
<=2	44	13.9
>2 and <=4	74	23.4
>4 and <=6	101	32.0
>6 and <=8	55	17.4
>8 and <=19	40	12.7
Unknown	2	0.6
Number of FTE nurses		
<=2	188	59.5
>2 and <=4	65	20.6
>4 and <=6	20	6.3
>6 and <=8	6	1.9
>8 and <=19	4	1.1
Unknown	33	10.4
QOF performance		
1st quintile (poorest performance)	50	15.8
2nd quintile	49	15.5
3rd quintile	59	18.7
4th quintile	82	26.0
5th quintile (best performance)	73	23.1
Unknown	3	1.0

GP consultations

The full model results for duration of GP consultations are given in Table S1 (Supplementary file). The following variables were excluded one by one from the full model: rate of nurse consultation (Z= 0.44, p=0.658), rurality (Z = 1.05, p=0.295), QOF performance (chi-squared=7.23, p=0.204), FTE nurses (chi-squared = 10.47, p=0.063), FTE GPs (chi-squared = 8.85, p=0.115), and list size (Z=-0.59, p=0.552). This yielded the final model in Table 3. Female patients' GP consultations were 8.3 seconds longer on average, and patients aged 0 to 14 years had shorter consultations than all other age groups. Those aged 45-64 had the longest consultations; consultations were 1.5 minutes longer, on average, than consultations in 0 to 14 year olds. Although both ethnic group (chi-squared = 85.2, p<0.001) and smoking status (chi-squared = 93.6, p<0.001) were retained in the model, only the unknown categories showed significant associations with duration of GP consultation: consultations with patients of unknown ethnicity were 11 seconds shorter than those with White patients, and consultations with patients of unknown smoking status were 19 seconds longer than those with non-smokers.

For peer review only - http://bmjopen.bmj.com/site/about/guidelines.xhtml

For peer review only

Table 3: Factors associated with duration of GP consultations

	Change in duration (seconds)	p-value	95% confidence interval	
Female gender (Male = reference)	8.29	0.000	6.03	10.55
Ethnic group (White = reference)				
Asian	4.06	0.237	-2.67	10.78
Chinese	-6.40	0.603	-30.49	17.69
Black	-5.70	0.200	-14.40	3.01
Mixed/ Other	4.30	0.289	-3.64	12.24
Unknown	-11.01	0.000	-13.51	-8.50
Index of multiple deprivation (1st quintile = reference)				
2nd quintile	1.14	0.537	-2.48	4.76
3rd quintile	-2.41	0.230	-6.35	1.53
4th quintile	-3.64	0.089	-7.83	0.56
5th quintile (most deprived)	-5.11	0.034	-9.84	-0.37
Unknown	-11.36	0.058	-23.12	0.39
Smoking status (Non-smoker = reference)				
Current smoker	-2.36	0.147	-5.54	0.83
Ex-smoker	0.11	0.943	-2.91	3.13
Unknown	18.65	0.000	14.56	22.73
Age group (0-14 years = reference)				
15-24	55.70	0.000	50.11	61.29
25-44	83.66	0.000	78.70	88.63
45-64	89.81	0.000	84.75	94.87
65-74	65.82	0.000	60.23	71.40
75+	58.43	0.000	52.94	63.92
Telephone consultation (Face-to-face = reference)	-308.71	0.000	-311.65	-305.77
Training practice (No = reference)				
Yes	44.33	0.000	19.87	68.78
Unknown	121.58	0.148	-43.02	286.17
GP consultation rate (centred, per 1000 per 10,000 person years)	-3.31	0.000	-4.24	-2.37
Mean duration	472.42	0.000	455.41	489.43

Duration of consultation decreased with increasing deprivation; consultations with patients in the most deprived quintile lasted 5 seconds less on average than consultations with the least deprived patients. Consultations in training practices were 44 seconds longer than those in practices that did not have trainee GPs, and, as expected, telephone consultations were, on average, 5 minutes shorter than face-to-face consultations. Finally, for every 10% increase in consultation rate (1000 per 10,000 person years), GP consultation duration decreased by 3 seconds.

Nurse consultations

The full model results for duration of nurse consultations are given in Table S2 (Supplementary file). Variables were removed from the full model as follows: ethnic group (chi-squared = 2.08, p=0.838), QOF performance (chi-squared = 2.57, p=0.767), training practice (Z=-0.61, p=0.544), rurality (Z=0.64, p=0.522), rate of GP consultation (Z=-0.60, p=0.547), FTE nurses (chi-squared = 6.24, p=0.284), and list size (Z=-1.15, p=0.250). The final model results are given in Table 4.

For peer review only - http://bmjopen.bmj.com/site/about/guidelines.xhtml

Table 4: Factors associated with duration of nurse consultations

	Change in duration (seconds)	p-value	95% confidence interval	
Female gender (Male = reference)	-11.06	0.000	-15.24	-6.88
Index of multiple deprivation (1st quintile = reference)				
2nd quintile	7.58	0.024	1.01	14.16
3rd quintile	-0.40	0.912	-7.56	6.75
4th quintile	5.49	0.158	-2.14	13.11
5th quintile (most deprived)	8.04	0.066	-0.53	16.62
Unknown	-26.93	0.007	-46.39	-7.46
Smoking status (Non-smoker = reference)				
Current smoker	26.67	0.000	20.80	32.55
Ex-smoker	15.20	0.000	9.80	20.60
Unknown	21.06	0.000	13.17	28.94
Age group (0-14 years = reference)				
15-24	52.30	0.000	41.70	62.91
25-44	71.85	0.000	62.54	81.16
45-64	113.15	0.000	103.70	122.59
65-74	73.81	0.000	63.71	83.90
75+	75.68	0.000	65.61	85.75
Telephone consultation (Face-to-face = reference)	-279.34	0.000	-288.18	-270.50
Number of FTE GPs (≤2 = reference)				
>2 and <=4	-85.82	0.004	-144.44	-27.20
>4 and <=6	-82.57	0.004	-138.53	-26.61
>6 and <=8	-82.90	0.008	-144.25	-21.55
>8 and <=19	-78.14	0.020	-143.96	-12.32
Unknown	-235.93	0.140	-549.15	77.29
Nurse consultation rate, centred, per 1000 per 10,000 person years)	-9.19	0.000	-11.53	-6.84
Mean duration	598.72	0.000	549.66	647.78

Consultations with a nurse were 11 seconds shorter for women than for men, and, in addition, all age groups had longer consultations than those aged 0 to 14 years (up to maximum of 2 minutes longer in those aged 45-64). Both current smokers and ex-smokers had longer nurse consultations than non-smokers, by an average of 27 and 15 seconds, respectively.

The effect of deprivation on duration of nurse consultation was unclear (overall chi-squared = 20.7, p=0.001). Those in the 2nd quintile had longer consultations than those in the least deprived quintile, but there was no clear relationship in other groups. Those with unknown deprivation had shorter consultations. In practices with more than 2 full-time equivalent GPs, nurse consultations were between 78 and 86 seconds shorter, although the effect of FTE GPs was marginally significant (chi-squared = 11.29, p=0.046), and was not significant when including list size in the model (chi-squared = 9.00, p=0.109). Practices with a higher rate of nurse consultation had shorter consultations by an average of 9 seconds for every 10% increase in consultation rate (1000 consultations per 10,000 person years).

DISCUSSION

We have shown that duration of consultation is associated with both patient and practice level characteristics, such as patient age and consultation rate. Telephone consultations, and those with a GP, are shorter than face-to-face consultations and those with a nurse. Patient age, patient gender,

For peer review only - http://bmjopen.bmj.com/site/about/guidelines.xhtml

and consultation rate are associated with duration of both GP and nurse consultations, although female gender increases the length of GP consultations and decreases the length of nurse consultations. Furthermore, GP consultations are longer in practices involved in GP training and with less deprived patients. Length of nurse consultations is not associated with patient deprivation or practice training status, but is increased in current and ex-smokers. Despite these associations, the observed differences in duration were small, indicating that consultation duration is not importantly higher or lower across different patient groups or general practices.

Strengths and limitations

This is a large-scale analysis of over 1 million consultations across England, and is therefore able to provide reliable estimates of association. Moreover, CPRD has been shown to be broadly representative of the UK population,[12] and hence our analysis is likely to be representative of those consulting across England. A further strength is our consideration of GP and nurse consultations separately in these analyses. This has allowed us to describe factors associated with the length of nurse consultations for the first time, and to compare results to those for GP consultations.

The duration of a consultation in CPRD reflects the length of time a patient record is open within the GP practice computer system, and is recorded in whole minutes. There were instances in the data of very long (>60 minutes) and very short (apparent 0 minutes) consultations which we rounded to 60 minutes and 0.5 minutes respectively. Long consultations may occur for genuine clinical need, but also if a staff member forgets to close the record at the end of the appointment. Short consultations may occur in the data if a record is opened incorrectly, or may have been included in this analysis if the type of consultation (e.g., administrative) was miscoded. Although duration may be recorded with some error, average durations were in line with a standard 10-minute appointment window, and extreme durations only affected 8.6% of all consultations studied, so we are confident this had little bearing on our results.

We did not have complete data on all individuals, and included some "Unknown" categories in our models, which may be difficult to interpret. Studies of the validity of smoking prevalence data in electronic health records suggest that former smoking is under-reported compared to UK national survey data,[13] so those with unknown smoking status in this study may be more likely to be former smokers. Ethnic group data was drawn from linked inpatient hospital episodes data, so those with missing data are likely to be healthier and consult less often, for less serious/complex conditions (ethnic group data was missing in 39% of patients, but only in 29% of consultations). This may explain why consultations in these patients were shorter as they may be less complex. Ethnicity data is similarly poorly reported in CPRD with fewer than 50% of patients ever having ethnicity recorded as of 2014,[12] hence, more detailed data is required to fully explore these associations.

We did not have data on the characteristics of individual nurses and GPs, so were unable to examine how such characteristics may affect duration. Previous research outside of the UK has shown that consultations with older GPs are longer[3,5] but, given the obvious link between practitioner age and experience, this contrasts with other UK-based research indicating that consultations are longer in those with lesser experience.[6] Our results regarding duration and practice training status are consistent with the UK research.

We did not examine the relationship between consultation duration and the number of presenting problems addressed in each appointment. A study of 229 GP consultations conducted in 2010 indicated that duration may be increased by 2 minutes for each additional presenting problem.[8] A

For peer review only - http://bmjopen.bmj.com/site/about/guidelines.xhtml

similar large-scale analysis using CPRD presents many methodological difficulties and is the subject of ongoing work by the study authors.

Comparison with the literature

Our findings are consistent with previous research indicating that increasing duration of GP consultation is associated with older patient age, [2–5,7] female gender,[3–7] and socioeconomic status.[5] We have also demonstrated that older patient age and current or prior smoking is associated with increased duration of nurse consultations. Although female patients have longer GP consultations, they have shorter nurse consultations, and the reason for this is unclear. One possible explanation is that nurses may conduct a larger proportion of relatively straightforward consultations with women (e.g. contraception reviews) compared to men. Although previous research has indicated that female patients have longer consultations with female GPs,[7] this study provides contemporary data from a much greater number of consultations.

We demonstrated that both GP and nurse consultations tend to be shorter in practices that have a greater corresponding consultation rate. This may indicate that GPs are limiting appointment lengths in order to meet consultation demand, and that there is little spare capacity in the appointment schedules. This is consistent with our previous work[1] which showed that overall GP workload has increased by 16% in England since 2007, and may be reaching saturation point. Conversely, more problems may be dealt with in a longer consultation, reducing the need for repeat consults. This was previously demonstrated by a study in two practices where increased initial consultation duration was associated with a lower consultation rate in the 4 weeks following the initial consultation.[14]

Implications

Our results indicate that only a handful of factors are associated with consultation duration, and overall, the absolute differences in duration are small. This suggests that, broadly speaking, patients are treated similarly regardless of their background, and consultation duration is equitable.

The largest observed differences were related to patient age, although this is likely to be confounded with comorbidity and complexity of consultation, which could not be directly measured in this study. Practices with an older or comorbid patient list could consider increasing the length of scheduled appointments to better match the required consultation time in these patients. Practices could also consider offering appointments of different lengths to different patients. Allowing patients to choose the length of their consultation has been shown to improve experiences for both patients and doctors, and patients are able, or could be educated, to estimate accurately their required consultations time.[15]

There is no strong evidence that consultation length is associated with patient satisfaction,[16,17] although increasing consultation duration has been shown to increase patient enablement, and decrease GP stress.[18] A previous review suggested that doctors conducting longer consultations are more likely to offer lifestyle or health promotion advice, and to recognise and deal with long-term problems.[19] Longer consultations may also reduce prescribing rates,[19] and be associated with more appropriate prescribing.[20] Evidence from exploratory trials also suggests that increasing consultation duration (as part of a wider complex intervention) is highly cost-effective.[21] However, a recent review indicated that many studies assessing interventions to alter consultation duration are at high risk of bias; the effect of altering duration on the number of referrals, prescriptions, or

For peer review only - http://bmjopen.bmj.com/site/about/guidelines.xhtml

patient satisfaction is uncertain.[22] Further research is required to establish the benefits and costs of increasing consultation duration alone.

We found that GP consultations are 44 seconds longer on average in GP practices hosting trainees, and this may have implications for the future of general practice. In recent years the recruitment of GPs has not kept pace with growth in the consulting population, and fewer trainees intend to stay in full time clinical work.[23] Policy makers and those responsible for recruitment should consider how the increased consultation time required to train GPs can be accommodated given increasing workload pressures.[1]

COMPETING INTERESTS

All authors have completed the ICMJE uniform disclosure form at www.icmje.org/coi_disclosure.pdf and declare: SS, CS and RP report grants from the National Institute for Health Research School for Primary Care Research, during the conduct of the study; no financial relationships with any organisations that might have an interest in the submitted work in the previous three years; no other relationships or activities that could appear to have influenced the submitted work.

FUNDING
This project is funded by the National Institute for Health Research School for Primary Care Research (NIHR SPCR). The views expressed are those of the author(s) and not necessarily those of the NHS, the NIHR or the Department of Health.

AUTHOR CONTRIBUTIONS
FDRH and CS conceived the research, obtained funding, and are joint principal investigators. FDRH, CB, and CS drafted the protocol, which all then contributed to. SS and TM were responsible for data management, and SS did the statistical analyses. SS drafted the report, which all authors then contributed to. SS is the guarantor and corresponding author.

DATA SHARING
Data is available from CPRD directly: www.cprd.com.

For peer review only

For peer review only - http://bmjopen.bmj.com/site/about/guidelines.xhtml

REFERENCES

1 Hobbs FDR, Bankhead C, Mukhtar T, *et al.* Clinical workload in UK primary care: a retrospective analysis of 100 million consultations in England, 2007–14. *Lancet* 2016;**387**:2323–30. doi:10.1016/S0140-6736(16)00620-6

2 van den Berg MJ, de Bakker DH, Wester GP, *et al.* Do list size and remuneration affect GPs' decisions about how they provide consultations? *BMC Health Serv Res* 2009;**9**. doi:10.1016/0277-9536(91)90087-S

3 Petek-Ster M, Svab I, Zivcec GK. Factors related to consultation time: experience in Slovenia. *Scand J Prim Health Care* 2008;**26**:29–34. doi:10.1080/02813430701760789

4 Deveugele M, Derese A, Brink-muinen A Van Den, *et al.* Consultation Length In General Practice: Cross Sectional Study. *BMJ* 2002;**325**:472–4.

5 Britt HC, Valenti L, Miller GC. Determinants of consultation length in Australian general practice. *Med J Aust* 2005;**183**:68–71.

6 Orton PK, Pereira Gray D. Factors influencing consultation length in general/family practice. *Fam Pract* 2016;**33**:529–34. doi:10.1093/fampra/cmw056

7 Carr-Hill R, Jenkins-Clarke S, Dixon P, *et al.* Do minutes count? Consultation lengths in general practice. *J Health Serv Res Policy* 1998;**3**:207–13.

8 Salisbury C, Procter S, Stewart K, *et al.* The content of general practice consultations: Cross-sectional study based on video recordings. *Br J Gen Pract* 2013;**63**:e751–9. doi:10.3399/bjgp13X674431

9 Health and Social Care Information Centre. General and personal medical services: England, 2004–2014, As at 30 September. 2015.http://content.digital.nhs.uk/catalogue/PUB16934 (accessed 11 May2015).

10 NHS Digital. Dataset: Rural/Urban definition of GP practice: categorical, 2011. 2011.https://indicators.hscic.gov.uk/webview/ (accessed 11 May2015).

11 Health and Social Care Information Centre. Quality and Outcomes Framework (QOF) - 2013-14. 2014.http://content.digital.nhs.uk/catalogue/PUB15751 (accessed 11 May2015).

12 Herrett E, Gallagher AM, Bhaskaran K, *et al.* Data Resource Profile: Clinical Practice Research Datalink (CPRD). *Int J Epidemiol* 2015;**44**:827–36. doi:10.1093/ije/dyv098

13 Booth HP, Prevost AT, Gulliford MC. Validity of smoking prevalence estimates from primary care electronic health records compared with national population survey data for England, 2007 to 2011. *Pharmacoepidemiol Drug Saf* 2013;**22**:1357–61. doi:10.1002/pds.3537

14 Hughes D. Consultation length and outcome in two group general practices. *J R Coll Gen Pract* 1983;**33**:143–7.

15 Sampson R, O'Rourke J, Hendry R, *et al.* Sharing control of appointment length with patients in general practice. *Br J Gen Pract* 2013;**63**:e185–91. doi:10.3399/bjgp13X668096

16 Elmore N, Burt J, Abel G, *et al.* Investigating the relationship between consultation length and patient experience: a cross-sectional study in primary care. *Br J Gen Pract* 2016;**66**.

17 Lemon TI, Smith RH. Consultation Content not Consultation Length Improves Patient Satisfaction. *J Fam Med Prim care* 2014;**3**:333–9. doi:10.4103/2249-4863.148102

18 Mercer SW, Fitzpatrick B, Gourlay G, *et al.* More time for complex consultations in a high-

For peer review only

For peer review only - http://bmjopen.bmj.com/site/about/guidelines.xhtml

deprivation practice is associated with increased patient enablement. *Br J Gen Pract* 2007;**57**:960–6. doi:10.3399/096016407782604910

19 Wilson A, Childs S. The effect of interventions to alter the consultation length of family physicians: A systematic review. *Br J Gen Pract* 2006;**56**:876–82.

20 Heaney DJ, Walker JJ, Howie JGR, *et al.* The development of a routine NHS data-based index of performance in general practice (NHSPPI). *Fam Pract* 2002;**19**:77–84.

21 Mercer SW, Fitzpatrick B, Guthrie B, *et al.* The CARE Plus study – a whole-system intervention to improve quality of life of primary care patients with multimorbidity in areas of high socioeconomic deprivation: exploratory cluster randomised controlled trial and cost-utility analysis. *BMC Med* 2016;**14**. doi:10.1136/bmj.h176

22 Wilson AD, Childs S, Gonçalves-Bradley DC, *et al.* Interventions to increase or decrease the length of primary care physicians' consultation. *Cochrane database Syst Rev* 2016;**8**:CD003540. doi:10.1002/14651858.CD003540.pub3

23 Baird B, Charles A, Honeyman M, *et al.* *Understanding pressures in general practice*. The Kings Fund 2016.

For peer review only

Supplement

Table S1: Full model for consultations with a GP

	Change in duration (seconds)	p-value	95% confidence interval	
Female gender (Male = reference)	8.29	0.000	6.03	10.55
Ethnic group (White = reference)				
Asian	4.11	0.231	-2.61	10.84
Chinese	-6.32	0.607	-30.41	17.76
Black	-5.63	0.205	-14.34	3.07
Mixed/ Other	4.35	0.283	-3.59	12.29
Unknown	-11.00	0.000	-13.50	-8.49
Index of multiple deprivation (1st quintile = reference)				
2nd quintile	1.14	0.535	-2.47	4.76
3rd quintile	-2.39	0.235	-6.33	1.55
4th quintile	-3.57	0.095	-7.77	0.62
5th quintile (most deprived)	-5.04	0.037	-9.77	-0.30
Unknown	-11.27	0.060	-23.02	0.48
Smoking status (Non-smoker = reference)				
Current smoker	-2.36	0.147	-5.54	0.83
Ex-smoker	0.11	0.944	-2.91	3.13
Unknown	18.66	0.000	14.58	22.75
Age group (0-14 years = reference)				
15-24	55.71	0.000	50.12	61.30
25-44	83.69	0.000	78.72	88.65
45-64	89.83	0.000	84.77	94.89
65-74	65.84	0.000	60.25	71.42
75+	58.44	0.000	52.96	63.93
Telephone consultation (Face-to-face = reference)	-308.70	0.000	-311.64	-305.76
Practice list size (centred, per 1000)	-5.21	0.026	-9.81	-0.61
Number of FTE GPs (≤2 = reference)				
>2 and <=4	27.74	0.206	-15.23	70.70
>4 and <=6	56.90	0.016	10.62	103.18
>6 and <=8	58.06	0.061	-2.61	118.73
>8 and <=19	120.02	0.002	43.58	196.47
Unknown	211.75	0.120	-54.93	478.44
Number of FTE nurses (≤2 = reference)				
>2 and <=4	-10.07	0.568	-44.59	24.46
>4 and <=6	-36.07	0.239	-96.11	23.97
>6 and <=8	-141.57	0.003	-235.44	-47.70
>8 and <=19	54.83	0.354	-61.14	170.81
Unknown	-16.52	0.429	-57.41	24.37
Training practice (No = reference)				
Yes	45.61	0.001	18.26	72.96
Unknown	-			
Nurse consultation rate (centred, per 1000 per 10,000 person years)	0.40	0.658	-1.36	2.15
GP consultation rate (centred, per 1000 per 10,000 person years)	-3.83	0.000	-4.80	-2.85
QOF performance (1st quintile = reference)				
2nd quintile	3.50	0.869	-38.00	44.99
3rd quintile	-16.22	0.427	-56.19	23.76
4th quintile	27.47	0.147	-9.67	64.61
5th quintile (best performance)	18.21	0.343	-19.40	55.83
Unknown	-50.43	0.633	-257.64	156.78
Rural practice (Urban = reference)	17.21	0.309	-15.98	50.39
Mean duration	417.27	0.000	367.50	467.04

Table S2: Full model for consultations with a nurse

	Change in duration (seconds)	p-value	95% confidence interval	
Female gender (Male = reference)	-11.15	0.000	-15.34	-6.96
Ethnic group (White = reference)				
Asian	0.18	0.979	-12.99	13.35
Chinese	-17.82	0.445	-63.57	27.93
Black	-1.67	0.849	-18.84	15.50
Mixed/ Other	-5.52	0.489	-21.18	10.13
Unknown	-2.52	0.279	-7.09	2.04
Index of multiple deprivation (1st quintile = reference)				
2nd quintile	7.49	0.026	0.92	14.06
3rd quintile	-0.54	0.883	-7.70	6.62
4th quintile	5.38	0.167	-2.24	13.01
5th quintile (most deprived)	7.92	0.071	-0.67	16.51
Unknown	-25.47	0.011	-45.10	-5.84
Smoking status (Non-smoker = reference)				
Current smoker	26.40	0.000	20.49	32.31
Ex-smoker	15.00	0.000	9.59	20.42
Unknown	21.12	0.000	13.23	29.01
Age group (0-14 years = reference)				
15-24	52.88	0.000	42.16	63.60
25-44	72.19	0.000	62.83	81.55
45-64	113.49	0.000	103.97	123.00
65-74	73.96	0.000	63.81	84.10
75+	75.54	0.000	65.43	85.65
Telephone consultation (Face-to-face = reference)	-279.51	0.000	-288.35	-270.66
Practice list size (centred, per 1000)	-6.09	0.065	-12.56	0.38
Number of FTE GPs (≤2 = reference)				
>2 and <=4	-63.99	0.045	-126.58	-1.39
>4 and <=6	-49.06	0.150	-115.82	17.70
>6 and <=8	-33.73	0.442	-119.68	52.22
>8 and <=19	-29.10	0.596	-136.61	78.42
Unknown	-106.02	0.625	-531.17	319.12
Number of FTE nurses (≤2 = reference)				
>2 and <=4	20.31	0.408	-27.79	68.41
>4 and <=6	49.99	0.240	-33.47	133.46
>6 and <=8	113.35	0.088	-16.77	243.48
>8 and <=19	137.25	0.096	-24.40	298.89
Unknown	-7.81	0.791	-65.58	49.97
Training practice (No = reference)				
Yes	-11.09	0.570	-49.31	27.14
Unknown	-			
Nurse consultation rate, centred, per 1000 per 10,000 person years)	-10.23	0.000	-12.84	-7.62
GP consultation rate, centred, per 1000 per 10,000 person years)	-0.49	0.488	-1.88	0.90
QOF performance (1st quintile = reference)				
2nd quintile	28.82	0.345	-30.93	88.56
3rd quintile	6.08	0.835	-50.99	63.14
4th quintile	9.11	0.738	-44.24	62.47
5th quintile (best performance)	31.22	0.254	-22.44	84.89
Unknown	-81.78	0.575	-367.98	204.42
Rural practice (Urban = reference)	13.93	0.561	-33.09	60.96
Mean duration	543.41	0.000	470.92	615.90

For peer review only

STROBE 2007 (v4) Statement—Checklist of items that should be included in reports of *cross-sectional studies*

Section/Topic	Item #	Recommendation	Reported on page #
**Title and abstract**	1	(*a*) Indicate the study's design with a commonly used term in the title or the abstract	1
		(*b*) Provide in the abstract an informative and balanced summary of what was done and what was found	2
**Introduction**			
Background/rationale	2	Explain the scientific background and rationale for the investigation being reported	3
Objectives	3	State specific objectives, including any prespecified hypotheses	3
**Methods**			
Study design	4	Present key elements of study design early in the paper	3
Setting	5	Describe the setting, locations, and relevant dates, including periods of recruitment, exposure, follow-up, and data collection	3
Participants	6	(*a*) Give the eligibility criteria, and the sources and methods of selection of participants	3
Variables	7	Clearly define all outcomes, exposures, predictors, potential confounders, and effect modifiers. Give diagnostic criteria, if applicable	4
Data sources/ measurement	8*	For each variable of interest, give sources of data and details of methods of assessment (measurement). Describe comparability of assessment methods if there is more than one group	3
Bias	9	Describe any efforts to address potential sources of bias	*3/4*
Study size	10	Explain how the study size was arrived at	3
Quantitative variables	11	Explain how quantitative variables were handled in the analyses. If applicable, describe which groupings were chosen and why	3
Statistical methods	12	(*a*) Describe all statistical methods, including those used to control for confounding	4
		(*b*) Describe any methods used to examine subgroups and interactions	N/A
		(*c*) Explain how missing data were addressed	4
		(*d*) If applicable, describe analytical methods taking account of sampling strategy	N/A
		(*e*) Describe any sensitivity analyses	N/A
**Results**			

For peer review only

Participants	13*	(a) Report numbers of individuals at each stage of study—eg numbers potentially eligible, examined for eligibility, confirmed eligible, included in the study, completing follow-up, and analysed	4
		(b) Give reasons for non-participation at each stage	4
		(c) Consider use of a flow diagram	N/A
Descriptive data	14*	(a) Give characteristics of study participants (eg demographic, clinical, social) and information on exposures and potential confounders	5/6
		(b) Indicate number of participants with missing data for each variable of interest	5/6
Outcome data	15*	Report numbers of outcome events or summary measures	5
Main results	16	(*a*) Give unadjusted estimates and, if applicable, confounder-adjusted estimates and their precision (eg, 95% confidence interval). Make clear which confounders were adjusted for and why they were included	7/8 and supplement
		(*b*) Report category boundaries when continuous variables were categorized	7/8
		(*c*) If relevant, consider translating estimates of relative risk into absolute risk for a meaningful time period	N/A
Other analyses	17	Report other analyses done—eg analyses of subgroups and interactions, and sensitivity analyses	N/A
**Discussion**			
Key results	18	Summarise key results with reference to study objectives	8
Limitations	19	Discuss limitations of the study, taking into account sources of potential bias or imprecision. Discuss both direction and magnitude of any potential bias	9
Interpretation	20	Give a cautious overall interpretation of results considering objectives, limitations, multiplicity of analyses, results from similar studies, and other relevant evidence	10
Generalisability	21	Discuss the generalisability (external validity) of the study results	10
**Other information**			
Funding	22	Give the source of funding and the role of the funders for the present study and, if applicable, for the original study on which the present article is based	11

*Give information separately for cases and controls in case-control studies and, if applicable, for exposed and unexposed groups in cohort and cross-sectional studies.

Note: An Explanation and Elaboration article discusses each checklist item and gives methodological background and published examples of transparent reporting. The STROBE checklist is best used in conjunction with this article (freely available on the Web sites of PLoS Medicine at http://www.plosmedicine.org/, Annals of Internal Medicine at http://www.annals.org/, and Epidemiology at http://www.epidem.com/). Information on the STROBE Initiative is available at www.strobe-statement.org.

For peer review only

Patient and practice level factors associated with consultation duration: a cross-sectional analysis of over 1 million consultations in English primary care.

Journal:	*BMJ Open*
Manuscript ID	bmjopen-2017-018261.R1
Article Type:	Research
Date Submitted by the Author:	24-Aug-2017
Complete List of Authors:	Stevens, Sarah; University of Oxford, Nuffield Department of Primary Care Health Sciences<br>Bankhead, Clare; University of Oxford<br>Mukhtar, Toqir; Nuffield Department of Primary Care Health Sciences<br>Perera, Rafael; University of Oxford, Primary Health Care<br>Holt, Tim; Oxford University, Department of Primary Care Health Sciences<br>Salisbury, Chris; University of Bristol, Academic Unit of Primary Health Care<br>Hobbs, Richard; University of Oxford, Dept of Primary Health Care
**Primary Subject Heading**:	General practice / Family practice
Secondary Subject Heading:	Health services research
Keywords:	PRIMARY CARE, Quality in health care < HEALTH SERVICES ADMINISTRATION & MANAGEMENT, Organisation of health services < HEALTH SERVICES ADMINISTRATION & MANAGEMENT, Health policy < HEALTH SERVICES ADMINISTRATION & MANAGEMENT

For peer review only

Patient and practice level factors associated with consultation duration: a cross-sectional analysis of over 1 million consultations in English primary care.

Sarah Stevens[1], Clare Bankhead[1], Toqir Mukhtar[1], Rafael Perera-Salazar[1], Tim Holt[1], Chris Salisbury[2], FD Richard Hobbs[1], on behalf of the NIHR School for Primary Care Research, Nuffield Department of Primary Care Health Sciences, University of Oxford

1. Nuffield Department of Primary Care Health Sciences, University of Oxford, Oxford, UK
2. Centre for Academic Primary Care, School of Social and Community Medicine, University of Bristol, Bristol, UK.

Corresponding author:

Sarah Stevens,

Contact details: sarah.stevens@phc.ox.ac.uk, 01865289449

Address: Nuffield Department of Primary Care Health Sciences, Radcliffe Primary Care Building, Radcliffe Observatory Quarter, Woodstock Road, Oxford, OX2 6GG;

Word count: 2998

For peer review only

ABSTRACT

Objectives: Consultation duration has previously been shown to be associated with patient, practitioner, and practice characteristics. However, previous studies were conducted outside the UK, considered only small numbers of GP consultations, or focused primarily on practitioner level characteristics. We aimed to determine the patient and practice level factors associated with duration of GP and nurse consultations in UK primary care.

Design and setting: Cross sectional data were obtained from English general practices contributing to the Clinical Practice Research Datalink (CPRD) linked to data on patient deprivation and practice staffing, rurality, and Quality and Outcomes Framework (QOF) achievement.

Participants: 218,304 patients, from 316 English general practices, consulting from 1st April 2013 to 31st March 2014.

Analysis: Multilevel mixed effects models described the association between consultation duration and patient and practice-level factors (patient age, gender, smoking status, ethnic group, deprivation and practice rurality, number of full time equivalent GPs/nurses, list size, consultation rate, quintile of overall QOF achievement, and training status).

Results: Mean duration of face-to-face GP consultations was 9.24 minutes and 5.32 minutes for telephone consultations. Nurse face-to-face and telephone consultations lasted 9.70 and 5.73 minutes on average, respectively. Longer GP consultation duration was associated with female patient gender, practice training status and older patient age. Shorter duration was associated with higher deprivation and consultation rate. Longer nurse consultation duration was associated with male patient gender, older patient age and ever smoking; and shorter duration with higher consultation rate. Observed differences in duration were small (e.g. GP consultations with female patients compared to male patients were 8 seconds longer on average).

Conclusions: Small observed differences in consultation duration indicate that patients are treated similarly regardless of background. Increased consultation duration may be beneficial for older or comorbid patients, but the benefits and costs of increased consultation duration require further study.

ARTICLE SUMMARY

Strengths and limitations of this study

- This is a large-scale analysis of over 1 million consultations, using data known to be representative of the UK population.
- We have considered factors associated with the duration of both GP and nurse consultations allowing comparison between the two.
- Appointment duration may be recorded with some error, but average durations were consistent with 10-minute appointment slots.
- We were unable to examine how GP / nurse characteristics are associated with consultation duration and this requires further study.

For peer review only

For peer review only - http://bmjopen.bmj.com/site/about/guidelines.xhtml

INTRODUCTION

Patient-facing general practice workload in England has increased by 16% since 2007.[1] This reflects an increase in both the rate and duration of consultations. Consultation duration may be influenced by patient, practitioner, and practice level characteristics. At the practice level, previous studies have shown that shorter consultation duration is associated with greater practice list size[2] and workload.[3] The influence of practice rurality (rural compared to urban) is unclear with some studies indicating that rurality is negatively associated with consultation duration[2,4], and others demonstrating a positive association.[5] Relevant practitioner characteristics associated with longer consultations include female gender,[6] older age[3,5], but conversely, lesser experience.[6] Finally, longer consultations have been shown to be associated with patient characteristics, including female gender,[3–5,7] older age,[2–5,7] greater number of presenting problems,[3–5,7,8] and higher level of education[3] or socioeconomic status.[5]

However, many previous studies have been conducted in countries other than the UK and findings may not be generalizable to the National Health Service.[2–5] Studies within the UK provide limited up-to-date evidence having been conducted some time ago using data on a relatively small number of consultations,[7] or having focused on practitioner level characteristics alone.[6] Although a 2013 paper studied the association between practice, practitioner, and patient level characteristics and the number of presenting problems, demonstrating that the number of presenting problems is also associated with consultation duration, direct links between patient and practice characteristics and duration were not studied.[8] Finally, previous work has considered duration of GP consultations only, despite nurse consultations accounting for approximately one quarter of the overall UK primary care consultation rate in 2013/14.[1] Hence, we aimed to determine the patient and practice characteristics associated with increased duration of GP and nurse consultations in UK primary care in contemporary data.

METHODS

Consultation and patient data were obtained from the Clinical Practice Research Datalink (CPRD), a research database of anonymised patient records drawn from over 600 UK general practices.[9] English practices consenting to CPRD's data linkage scheme were included in the study if they contributed data covering any part of the study period (1st April 2013 to 31st March 2014), and were defined as "up-to-standard" (CPRD definition of continuous high quality data recording fit for use in research). All non-temporary patients registered at eligible practices for at least one day during the study period were included. Due to data volume, analysis was limited to a 10% simple random sample from each age-sex strata of eligible patents and those who consulted at least once during the study period.

CPRD data was linked to practice data on staffing,[10] rurality,[11] and Quality and Outcomes Framework (QOF) performance measures,[12] and patient Index of Multiple Deprivation (IMD). IMD data was supplied in quintiles by CPRD, who link patient postcodes to publically available IMD scores and group data into quintiles at the English national level. Staffing, rurality, and QOF data was downloaded from NHS digital (formerly the Health and Social Care Information Centre), and continuous variables were grouped prior to linkage with CPRD data. This was a requirement of the Independent Scientific Advisory Committee (ISAC) to CPRD, to limit the possibility of identifying individual CPRD practices. The approved protocol (number 15_120R) is available from the authors.

Consultations in CPRD represent occasions on which a patient's electronic health record is opened. We analysed consultations that were identified as face-to-face or telephone consultations based on

For peer review only - http://bmjopen.bmj.com/site/about/guidelines.xhtml

the variable “consultation type”, and those with a GP or nurse only, as indicated by the variable “staff role”. We excluded consultations where the patient record was opened purely for administrative purposes by GPs, nurses, or administrative staff (e.g. to record test results) and home visit consultations (since recorded duration may merely represent the time taken to record the consultation after it has ended).

Mean consultation duration across practices was examined using histograms. Practices were grouped according to their average consultation duration (<5, ≥5 and <8, ≥8 and <10, ≥10 and <12, ≥12 and <15 and ≥15 minutes) and differences in their characteristics described.

Multilevel mixed effects models were used to model the association between patient and practice characteristics and duration of GP or nurse consultations separately. Patient factors included as fixed effects were age, gender, smoking status (current, former, never), ethnic group, and quintile of IMD. Fixed effects practice level factors included were rurality, number of full time equivalent (FTE) GPs, number of FTE nurses, list size (centred), rate of GP consultation (centred), rate of nurse consultation (centred), quintile of overall QOF achievement, and practice training status (yes or no). Indicators for the patient and practice were included as random effects. All variables were entered into the models simultaneously and subsequently excluded in a stepwise fashion based on Z tests (binary and continuous variables), or chi-squared tests (categorical variables) at the 5% level. Missing smoking status and ethnic group data were included as separate categories in the models.

RESULTS

In total, 3,049,320 patients were eligible during the study period, of which 304,937 were randomly selected for inclusion. Of these, 218,304 consulting patients from 316 practices were included. The characteristics of the included patients and practices are given in Table 1 and Table 2. During the study period 964,148 consultations were conducted by a GP, and 347,657 were conducted by a nurse. The majority of consultations (1,155,040; 88%) were face-to-face consultations. Mean duration of face-to-face GP consultations was 9.24 (SD=8.06) minutes compared to 5.32 (6.21) minutes for telephone consultations. Nurse consultations were longer, on average, than those with GPs; face-to-face and telephone nurse consultations lasted 9.70 (9.21) and 5.73 (6.29) minutes. A minority of practices conducted substantially shorter or longer consultations on average (Figure S1).

For peer review only

Table 1: Characteristics of included patients (N=218,304)

	Mean/ N	SD/ %
Female gender	121,107	55.5
Age group		
0-14 years	36,371	16.7
15-24	23,020	10.5
25-44	55,316	25.3
45-64	57,000	26.1
65-74	24,086	11.0
75+	22,511	10.3
Smoking status		
Non-smoker	82,327	37.7
Current smoker	37,286	17.1
Ex-smoker	40,834	18.7
Unknown	57,857	26.5
Index of multiple deprivation		
1st quintile (least deprived)	48,363	22.2
2nd quintile	47,948	22.0
3rd quintile	41,825	19.2
4th quintile	41,953	19.2
5th quintile (most deprived)	34,750	15.9
Unknown	3,465	1.6
Ethnic group		
White	118,063	54.1
Asian	6,008	2.8
Chinese	491	0.2
Black	3,908	1.8
Mixed/ Other	4,374	2.0
Unknown	85,460	39.2

For peer review only

Table 2: Characteristics of included practices (N=316)

	Mean/ N	SD/ %
List size	9,649.7	4,648.4
Training practice		
Yes	126	39.9
Unknown	2	0.6
Rurality		
Not rural (Urban >10K - less sparse)	267	84.5
Rural (Hamlet/village/town & fringe)	49	15.5
GP consultation rate (per 10,000 person years)	37,441.0	13,043.6
Nurse consultation rate (per 10,000 person years)	13,217.3	7,580.5
Number of FTE GPs		
<=2	44	13.9
>2 and <=4	74	23.4
>4 and <=6	101	32.0
>6 and <=8	55	17.4
>8 and <=19	40	12.7
Unknown	2	0.6
Number of FTE nurses		
<=2	188	59.5
>2 and <=4	65	20.6
>4 and <=6	20	6.3
>6 and <=8	6	1.9
>8 and <=19	4	1.1
Unknown	33	10.4
QOF performance		
1st quintile (poorest performance)	50	15.8
2nd quintile	49	15.5
3rd quintile	59	18.7
4th quintile	82	26.0
5th quintile (best performance)	73	23.1
Unknown	3	1.0

GP consultations

Practice characteristics by average length of GP consultation, are described in Table S1. Practices conducting longer consultations had a lower rate of GP consultation but the relationship between other characteristics was less clear. Full model results for duration of GP consultations are given in Table S2 and variables were excluded in the following order: rate of nurse consultation (p=0.658), rurality (p=0.295), QOF performance (p=0.204), FTE nurses (p=0.063), FTE GPs (p=0.115), and list size (p=0.552). This yielded the final model in Table 3. Female patients' GP consultations were 8.3 seconds longer on average, and patients aged 0 to 14 years had the shortest consultations. Those aged 45-64 had the longest consultations; consultations were 1.5 minutes longer, on average, than consultations in 0 to 14 year olds. Although both ethnic group (p<0.001) and smoking status (p<0.001) were retained in the model, only the unknown categories showed significant associations: consultations with patients of unknown ethnicity were 11 seconds shorter than those with White patients, and consultations with patients of unknown smoking status were 19 seconds longer than those with non-smokers.

For peer review only - http://bmjopen.bmj.com/site/about/guidelines.xhtml

Table 3: Factors associated with duration of GP consultations

	Change in duration (seconds)	p-value	95% confidence interval	
Female gender (Male = reference)	8.29	0.000	6.03	10.55
Ethnic group (White = reference)				
Asian	4.06	0.237	-2.67	10.78
Chinese	-6.40	0.603	-30.49	17.69
Black	-5.70	0.200	-14.40	3.01
Mixed/ Other	4.30	0.289	-3.64	12.24
Unknown	-11.01	0.000	-13.51	-8.50
Index of multiple deprivation (1st quintile = reference)				
2nd quintile	1.14	0.537	-2.48	4.76
3rd quintile	-2.41	0.230	-6.35	1.53
4th quintile	-3.64	0.089	-7.83	0.56
5th quintile (most deprived)	-5.11	0.034	-9.84	-0.37
Unknown	-11.36	0.058	-23.12	0.39
Smoking status (Non-smoker = reference)				
Current smoker	-2.36	0.147	-5.54	0.83
Ex-smoker	0.11	0.943	-2.91	3.13
Unknown	18.65	0.000	14.56	22.73
Age group (0-14 years = reference)				
15-24	55.70	0.000	50.11	61.29
25-44	83.66	0.000	78.70	88.63
45-64	89.81	0.000	84.75	94.87
65-74	65.82	0.000	60.23	71.40
75+	58.43	0.000	52.94	63.92
Telephone consultation (Face-to-face = reference)	-308.71	0.000	-311.65	-305.77
Training practice (No = reference)				
Yes	44.33	0.000	19.87	68.78
Unknown	121.58	0.148	-43.02	286.17
GP consultation rate (centred, per 1000 per 10,000 person years)	-3.31	0.000	-4.24	-2.37
Mean duration	472.42	0.000	455.41	489.43

Duration of consultation decreased with increasing deprivation; consultations with patients in the most deprived quintile lasted 5 seconds less on average than consultations with the least deprived patients. Consultations in training practices were 44 seconds longer than those in practices that did not have trainee GPs, and telephone consultations were, on average, 5 minutes shorter than face-to-face consultations. Finally, for every 10% increase in consultation rate (1000 per 10,000 person years), GP consultation duration decreased by 3 seconds.

In post-hoc sensitivity analysis, we explored whether the association of duration with practice training status may be driven by consultations with trainee GPs alone, by adding a variable into the final model to indicate whether the GP conducting the consultation was a registrar or not. We found that consultations were on average 245 seconds longer with a GP registrar than otherwise and practice training status became non-significant (p=0.0656, Table S3).

Nurse consultations

Practice characteristics, by average length of nurse consultation, are described in Table S4. Similarly to GP consultations, practices conducting longer consultations had a lower rate of nurse

For peer review only

consultation. Full model results for duration of nurse consultations are given in Table S5. Variables were removed from the full model as follows: ethnic group (p=0.838), QOF performance (p=0.767), training practice (p=0.544), rurality (p=0.522), rate of GP consultation (p=0.547), FTE nurses (p=0.284), and list size (p=0.250).

In the final model (Table 4) consultations with a nurse were 11 seconds shorter for women than for men. All age groups had longer consultations than those aged 0 to 14 years (up to maximum of 2 minutes longer in those aged 45-64). Current smokers and ex-smokers had longer nurse consultations than non-smokers, by an average of 27 and 15 seconds, respectively. Those in the 2nd quintile of deprivation had longer consultations than those in the least deprived quintile, but there was no clear relationship in other groups. Those with unknown deprivation had shorter consultations. In practices with more than two FTE GPs, nurse consultations were between 78 and 86 seconds shorter, although the effect of FTE GPs was marginally significant (p=0.046), and was not significant when including list size in the model (p=0.109). Practices with a higher rate of nurse consultation had shorter consultations by an average of 9 seconds for every 10% increase in consultation rate (1000 consultations per 10,000 person years).

Table 4: Factors associated with duration of nurse consultations

	Change in duration (seconds)	p-value	95% confidence interval	
Female gender (Male = reference)	-11.06	0.000	-15.24	-6.88
Index of multiple deprivation (1st quintile = reference)				
2nd quintile	7.58	0.024	1.01	14.16
3rd quintile	-0.40	0.912	-7.56	6.75
4th quintile	5.49	0.158	-2.14	13.11
5th quintile (most deprived)	8.04	0.066	-0.53	16.62
Unknown	-26.93	0.007	-46.39	-7.46
Smoking status (Non-smoker = reference)				
Current smoker	26.67	0.000	20.80	32.55
Ex-smoker	15.20	0.000	9.80	20.60
Unknown	21.06	0.000	13.17	28.94
Age group (0-14 years = reference)				
15-24	52.30	0.000	41.70	62.91
25-44	71.85	0.000	62.54	81.16
45-64	113.15	0.000	103.70	122.59
65-74	73.81	0.000	63.71	83.90
75+	75.68	0.000	65.61	85.75
Telephone consultation (Face-to-face = reference)	-279.34	0.000	-288.18	-270.50
Number of FTE GPs (≤2 = reference)				
>2 and <=4	-85.82	0.004	-144.44	-27.20
>4 and <=6	-82.57	0.004	-138.53	-26.61
>6 and <=8	-82.90	0.008	-144.25	-21.55
>8 and <=19	-78.14	0.020	-143.96	-12.32
Unknown	-235.93	0.140	-549.15	77.29
Nurse consultation rate, centred, per 1000 per 10,000 person years)	-9.19	0.000	-11.53	-6.84
Mean duration	598.72	0.000	549.66	647.78

For peer review only - http://bmjopen.bmj.com/site/about/guidelines.xhtml

DISCUSSION

We have shown that duration of consultation is associated with both patient and practice level characteristics. Increasing patient age is associated with increased consultation duration. Female patient gender increases the length of GP consultations and decreases the length of nurse consultations and duration of nurse consultations is increased in current and ex-smokers. GP consultations are longer in practices involved in GP training and with less deprived patients, but shorter in practices with a higher consultation rate. Although there is some variation in mean duration across practices, this is not explained by many of the practice characteristics studied.

Strengths and limitations

This is a large-scale analysis of over 1 million consultations across England, and is therefore able to provide reliable estimates of association. Moreover, CPRD has been shown to be broadly representative of the UK population,[9] and our results are likely to be representative of those consulting across England. A further strength is our separate consideration of GP and nurse consultations, allowing us to describe factors associated with the length of nurse consultations for the first time. A limitation is the consideration of consultations occurring in general practice only and our results may not be generalizable to other settings (e.g. walk in centres).

Consultation duration in CPRD reflects the length of time a patient record is open within the practice computer system, recorded in whole minutes. There were instances in the data of very long (>60 minutes, 0.3%) and very short (apparent 0 minutes, 8.3%) consultations which we rounded to 60 minutes and 0.5 minutes respectively. Long consultations may occur for genuine clinical need, but also if a staff member forgets to close a record. Apparent short consultations may occur if a record is opened incorrectly, if details of a straightforward consultation are entered only at the end of a consultation, or if the type of consultation (e.g. administrative) was miscoded. However, average durations were in line with a standard 10-minute appointment window, and final model estimates were similar when excluding these extreme durations or including them without rounding (data not shown).

Due to missing data, we included some “Unknown” categories in our models. Previous research has shown that former smoking is under-reported in CPRD compared to UK national survey data,[13] so those with unknown status in this study may be more likely to be former smokers. Ethnic group data was drawn from hospital episodes data, so those with missing data may be healthier and consult less often (ethnic group was missing in 39% of patients, but only in 29% of consultations). Hence consultations in these patients may have been shorter and less complex. Ethnicity data is similarly poorly reported in CPRD,[9] hence, more detailed data is required to fully explore these associations.

We did not have data on the characteristics of nurses and GPs, so were unable to examine their association with duration. Previous research outside of the UK has shown that consultations with older GPs are longer,[3,5] but this contrasts with UK-based research indicating that consultations are longer in those with lesser experience.[6] Our primary/ post-hoc results regarding the association of duration with practice training status/ GP registrars are consistent with the UK research. However, we found that in practices which were not identified as training practices in the national data, 4.9% of GP consultations appeared to be conducted by GP registrars (compared to 11.6% in training practices). This indicates inaccuracies in coding either of staff role or of training practice status, and hence this finding needs further replication in future studies.

We did not examine the relationship between consultation duration and the number of presenting problems. A 2010 study indicated that GP consultation duration may be increased by 2 minutes for

For peer review only - http://bmjopen.bmj.com/site/about/guidelines.xhtml

each additional presenting problem.[8] A similar large-scale analysis using CPRD presents many methodological difficulties and is the subject of ongoing work by the study authors.

Comparison with the literature

Our contemporary results confirm previous research findings that increasing duration of GP consultation is associated with older patient age, [2–5,7] female patient gender,[3–7] and socioeconomic status.[5] Older patient age and current or prior smoking are also associated with increased duration of nurse consultations. Although female patients have longer GP consultations, they have shorter nurse consultations, and the reason for this is unclear. Nurses may conduct more relatively straightforward consultations with women (e.g. contraception reviews) compared to with men.

GP and nurse consultations are shorter in practices with a greater corresponding consultation rate, perhaps indicating that appointment lengths are limited to meet consultation demand, with little spare capacity in schedules. This is consistent with our previous work[1] showing that GP workload has increased by 16% in England since 2007,[1] and may be reaching saturation point. Conversely, more problems may be dealt with in a longer consultation, reducing the need for repeat consults. This was previously demonstrated by a study in two practices where increased initial consultation duration was associated with a lower consultation rate in the following 4 weeks.[14]

Implications

We observed small absolute differences in consultation duration, despite statistical significance for some factors. This may suggest that all patients are treated similarly and that consultation duration is equitable and in line with patient need. For example we observed large differences related to patient age, which is likely to be confounded with comorbidity and complexity of consultation. However, our findings that more deprived patients have shorter consultations on average could indicate inequalities based on clinical need since more deprived patients have higher rates of premature mortality.[15] Practices with an older or comorbid patient list could increase the length of scheduled appointments to better match the required consultation time in these patients. Practices could also allow patients to choose their consultation length. This has been shown to improve doctor and patient experience, and patients could be educated to estimate their required time.[16]

We observed a small decrease of three seconds in the duration of GP consultations for every 10% increase in the rate of consultation, which is unlikely to be clinically important. However, increases in consultation rates above 10% could negatively impact clinical care. The importance of consultation duration partly depends on whether longer consultations are associated with improved outcomes. Increasing duration has been shown to increase patient enablement, and decrease GP stress.[17] A previous review suggested that doctors conducting longer consultations are more likely to offer health promotion advice, and deal with long-term problems.[18] Longer consultations may also reduce prescribing rates,[18] and be associated with more appropriate prescribing.[19] However there is little strong evidence that consultation length is associated with patient satisfaction generally,[18,20] or when GPs are pre-selected for poor communication,[21] although one study has shown an association with more patient centeredness.[6]

Evidence from exploratory trials suggests that increasing consultation duration (as part of a wider complex intervention) is highly cost-effective.[22] However, a recent review indicated that many studies assessing interventions to alter consultation duration are at high risk of bias; the effect of altering duration on the number of referrals, prescriptions, or patient satisfaction is uncertain.[23]

For peer review only - http://bmjopen.bmj.com/site/about/guidelines.xhtml

Further research is required to establish the benefits and costs of increasing consultation duration alone.

GP consultations are longer on average in practices hosting trainees. This may have implications for the future of general practice since GP recruitment has not kept pace with growth in the consulting population, and fewer trainees intend to stay in full time clinical work.[24] Policy makers and those responsible for recruitment should consider how the increased time required to train GPs can be accommodated given increasing workload pressures.[1]

COMPETING INTERESTS

All authors have completed the ICMJE uniform disclosure form at www.icmje.org/coi_disclosure.pdf and declare: SS, CS and RP report grants from the National Institute for Health Research School for Primary Care Research, during the conduct of the study; no financial relationships with any organisations that might have an interest in the submitted work in the previous three years; no other relationships or activities that could appear to have influenced the submitted work.

FUNDING

This project is funded by the National Institute for Health Research School for Primary Care Research (NIHR SPCR). FDRH is partly supported as a NIHR Senior Investigator, Director of the NIHR SPCR, Director of the NIHR CLARHC Oxford, Theme Leader of the NIHR Oxford BRC, NIHR Oxford DEC, and as a Professorial Fellow of Harris Manchester College. The views expressed are those of the author(s) and not necessarily those of the NHS, the NIHR or the Department of Health.

AUTHOR CONTRIBUTIONS

FDRH and CS conceived the research, obtained funding, and are joint principal investigators. FDRH, CB, and CS drafted the protocol, which all then contributed to. SS and TM were responsible for data management, and SS did the statistical analyses. SS drafted the report, which all authors then contributed to. SS is the guarantor and corresponding author.

DATA SHARING

Data is available from CPRD directly: www.cprd.com.

For peer review only

For peer review only - http://bmjopen.bmj.com/site/about/guidelines.xhtml

REFERENCES

1 Hobbs FDR, Bankhead C, Mukhtar T, *et al.* Clinical workload in UK primary care: a retrospective analysis of 100 million consultations in England, 2007–14. *Lancet* 2016;**387**:2323–30. doi:10.1016/S0140-6736(16)00620-6

2 van den Berg MJ, de Bakker DH, Wester GP, *et al.* Do list size and remuneration affect GPs' decisions about how they provide consultations? *BMC Health Serv Res* 2009;**9**. doi:10.1016/0277-9536(91)90087-S

3 Petek-Ster M, Svab I, Zivcec GK. Factors related to consultation time: experience in Slovenia. *Scand J Prim Health Care* 2008;**26**:29–34. doi:10.1080/02813430701760789

4 Deveugele M, Derese A, Brink-muinen A Van Den, *et al.* Consultation Length In General Practice: Cross Sectional Study. *BMJ* 2002;**325**:472–4.

5 Britt HC, Valenti L, Miller GC. Determinants of consultation length in Australian general practice. *Med J Aust* 2005;**183**:68–71.

6 Orton PK, Pereira Gray D. Factors influencing consultation length in general/family practice. *Fam Pract* 2016;**33**:529–34. doi:10.1093/fampra/cmw056

7 Carr-Hill R, Jenkins-Clarke S, Dixon P, *et al.* Do minutes count? Consultation lengths in general practice. *J Health Serv Res Policy* 1998;**3**:207–13.

8 Salisbury C, Procter S, Stewart K, *et al.* The content of general practice consultations: Cross-sectional study based on video recordings. *Br J Gen Pract* 2013;**63**:e751–9. doi:10.3399/bjgp13X674431

9 Herrett E, Gallagher AM, Bhaskaran K, *et al.* Data Resource Profile: Clinical Practice Research Datalink (CPRD). *Int J Epidemiol* 2015;**44**:827–36. doi:10.1093/ije/dyv098

10 Health and Social Care Information Centre. General and personal medical services: England, 2004–2014, As at 30 September. 2015.http://content.digital.nhs.uk/catalogue/PUB16934 (accessed 11 May2015).

11 NHS Digital. Dataset: Rural/Urban definition of GP practice: categorical, 2011. 2011.https://indicators.hscic.gov.uk/webview/ (accessed 11 May2015).

12 Health and Social Care Information Centre. Quality and Outcomes Framework (QOF) - 2013-14. 2014.http://content.digital.nhs.uk/catalogue/PUB15751 (accessed 11 May2015).

13 Booth HP, Prevost AT, Gulliford MC. Validity of smoking prevalence estimates from primary care electronic health records compared with national population survey data for England, 2007 to 2011. *Pharmacoepidemiol Drug Saf* 2013;**22**:1357–61. doi:10.1002/pds.3537

14 Hughes D. Consultation length and outcome in two group general practices. *J R Coll Gen Pract* 1983;**33**:143–7.

15 Kontopantelis E, Springate DA, Ashworth M, *et al.* Investigating the relationship between quality of primary care and premature mortality in England: a spatial whole-population study. *BMJ* 2015;**350**:h904.

16 Sampson R, O'Rourke J, Hendry R, *et al.* Sharing control of appointment length with patients in general practice. *Br J Gen Pract* 2013;**63**:e185–91. doi:10.3399/bjgp13X668096

17 Mercer SW, Fitzpatrick B, Gourlay G, *et al.* More time for complex consultations in a high-deprivation practice is associated with increased patient enablement. *Br J Gen Pract* 2007;**57**:960–6. doi:10.3399/096016407782604910

For peer review only - http://bmjopen.bmj.com/site/about/guidelines.xhtml

18 Wilson A, Childs S. The effect of interventions to alter the consultation length of family physicians: A systematic review. *Br J Gen Pract* 2006;**56**:876–82.

19 Heaney DJ, Walker JJ, Howie JGR, *et al.* The development of a routine NHS data-based index of performance in general practice (NHSPPI). *Fam Pract* 2002;**19**:77–84.

20 Lemon TI, Smith RH. Consultation Content not Consultation Length Improves Patient Satisfaction. *J Fam Med Prim care* 2014;**3**:333–9. doi:10.4103/2249-4863.148102

21 Elmore N, Burt J, Abel G, *et al.* Investigating the relationship between consultation length and patient experience: a cross-sectional study in primary care. *Br J Gen Pract* 2016;**66**.

22 Mercer SW, Fitzpatrick B, Guthrie B, *et al.* The CARE Plus study – a whole-system intervention to improve quality of life of primary care patients with multimorbidity in areas of high socioeconomic deprivation: exploratory cluster randomised controlled trial and cost-utility analysis. *BMC Med* 2016;**14**. doi:10.1136/bmj.h176

23 Wilson AD, Childs S, Gonçalves-Bradley DC, *et al.* Interventions to increase or decrease the length of primary care physicians' consultation. *Cochrane database Syst Rev* 2016;**8**:CD003540. doi:10.1002/14651858.CD003540.pub3

24 Baird B, Charles A, Honeyman M, *et al. Understanding pressures in general practice*. The Kings Fund 2016.

For peer review only

For peer review only - http://bmjopen.bmj.com/site/about/guidelines.xhtml

Supplement

Figure S1: Distribution of mean consultation duration in each practice for face-to-face or telephone consultations conducted by a GP or nurse

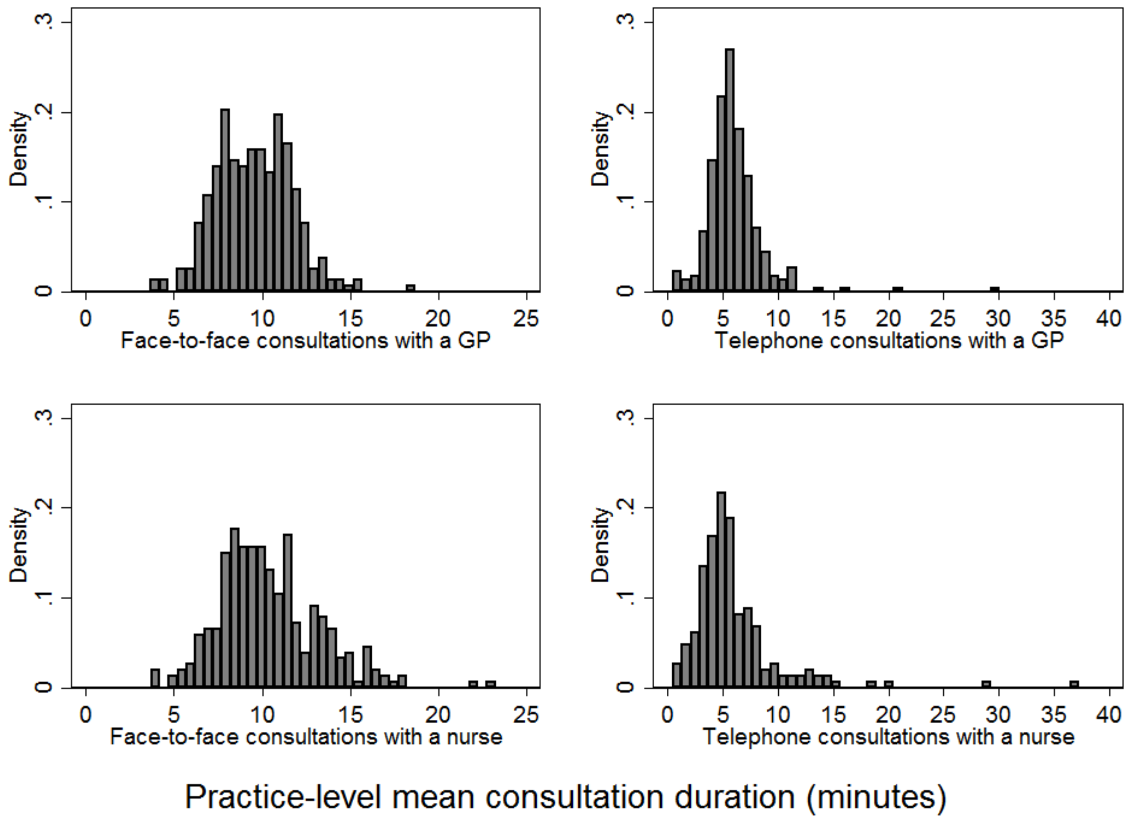

For peer review only

Table S1: Characteristics of practices according to mean duration of consultation with a GP

	Practice mean consultation duration (minutes) (N=316)					
	<5	≥5 and <8	≥8 and <10	≥10 and <12	≥12 and <15	≥15
**Number of practices (%)**	**4 (1.3)**	**96 (30.4)**	**118 (37.3)**	**83 (26.3)**	**14 (4.4)**	**1 (0.3)**
Mean list size (SD)	5,580.0 (713.0)	10,149.3 (4,603.6)	10,232.1 (4809.5)	8,771.6 (4,590.9)	7,715.2 (2,912.3)	9,220.0 (-)
Training practice						
Yes: N (%)	0 (0.0)	33 (34.4)	53 (44.9)	35 (42.2)	5 (35.7)	0 (0.0)
Rurality						
Not rural (Urban >10K - less sparse):N (%)	4 (100.0)	82 (85.4)	101 (85.6)	68 (81.9)	12 (85.7)	0 (0.0)
Mean GP consultation rate (per 10,000 person years) (SD)	46,877.5 (17,478.6)	42,488.2 (16,711.8)	37,671.8 (9,954.9)	32,618.1 (9,227.8)	28,463.9 (9,387.5)	13,895.5 (-)
Mean nurse consultation rate (per 10,000 person years) (SD)	12,777.7 (4,657.8)	14,270.9 (7,893.9)	13,566.1 (7,677.1)	11,707.4 (6,888.7)	12,279.2 (8,778.4)	11,124.7 (-)
Number of FTE GPs						
<=2: N (%)	2 (50.0)	9 (9.4)	16 (13.6)	14 (16.9)	3 (21.4)	0 (0.0)
>2 and <=4: N (%)	2 (50.0)	24 (25.0)	22 (18.6)	24 (28.9)	2 (14.3)	0 (0.0)
>4 and <=6: N (%)	0 (0.0)	37 (38.5)	32 (27.1)	24 (28.9)	7 (50.0)	1 (100.0)
>6 and <=8: N (%)	0 (0.0)	15 (15.6)	25 (21.2)	15 (18.1)	0 (0.0)	0 (0.0)
>8 and <=19: N (%)	0 (0.0)	11 (11.5)	22 (18.6)	5 (6.0)	2(14.3)	0 (0.0)
Number of FTE nurses						
<=2: N (%)	4 (100.0)	51 (53.1)	69 (58.5)	52 (62.7)	12 (85.7)	0 (0.0)
>2 and <=4: N (%)	0 (0.0)	22 (22.9)	22 (18.6)	18 (21.7)	2 (14.3)	1 (100.0)
>4 and <=6: N (%)	0 (0.0)	8 (8.3)	10 (8.5)	2 (2.4)	0 (0.0)	0 (0.0)
>6 and <=8: N (%)	0 (0.0)	4 (4.2)	2 (1.7)	0 (0.0)	0 (0.0)	0 (0.0)
>8 and <=19: N (%)	0 (0.0)	2 (2.1)	2 (1.7)	0 (0.0)	0 (0.0)	0 (0.0)
QOF performance						
1st quintile (poorest performance): N (%)	2 (50.0)	15 (15.6)	18 (15.3)	12 (14.5)	3 (21.4)	0 (0.0)
2nd quintile: N (%)	0 (0.0)	22(22.9)	13 (11.0)	13 (15.7)	1 (7.1)	0 (0.0)
3rd quintile: N (%)	1 (25.0)	23 (24.0)	24 (20.3)	8 (9.6)	2 (14.3)	1 (100.0)
4th quintile: N (%)	1 (25.0)	18 (18.8)	31 (26.3)	27 (32.5)	5 (35.7)	0 (0.0)
5th quintile (best performance): N (%)	0 (0.0)	18 (18.8)	30 (25.4)	22 (26.5)	3 (21.4)	0 (0.0)

For peer review only

Table S2: Full model for consultations with a GP

	Change in duration (seconds)	p-value	95% confidence interval	
Female gender (Male = reference)	8.29	0.000	6.03	10.55
Ethnic group (White = reference)				
Asian	4.11	0.231	-2.61	10.84
Chinese	-6.32	0.607	-30.41	17.76
Black	-5.63	0.205	-14.34	3.07
Mixed/ Other	4.35	0.283	-3.59	12.29
Unknown	-11.00	0.000	-13.50	-8.49
Index of multiple deprivation (1st quintile = reference)				
2nd quintile	1.14	0.535	-2.47	4.76
3rd quintile	-2.39	0.235	-6.33	1.55
4th quintile	-3.57	0.095	-7.77	0.62
5th quintile (most deprived)	-5.04	0.037	-9.77	-0.30
Unknown	-11.27	0.060	-23.02	0.48
Smoking status (Non-smoker = reference)				
Current smoker	-2.36	0.147	-5.54	0.83
Ex-smoker	0.11	0.944	-2.91	3.13
Unknown	18.66	0.000	14.58	22.75
Age group (0-14 years = reference)				
15-24	55.71	0.000	50.12	61.30
25-44	83.69	0.000	78.72	88.65
45-64	89.83	0.000	84.77	94.89
65-74	65.84	0.000	60.25	71.42
75+	58.44	0.000	52.96	63.93
Telephone consultation (Face-to-face = reference)	-308.70	0.000	-311.64	-305.76
Practice list size (centred, per 1000)	-5.21	0.026	-9.81	-0.61
Number of FTE GPs (≤2 = reference)				
>2 and <=4	27.74	0.206	-15.23	70.70
>4 and <=6	56.90	0.016	10.62	103.18
>6 and <=8	58.06	0.061	-2.61	118.73
>8 and <=19	120.02	0.002	43.58	196.47
Unknown	211.75	0.120	-54.93	478.44
Number of FTE nurses (≤2 = reference)				
>2 and <=4	-10.07	0.568	-44.59	24.46
>4 and <=6	-36.07	0.239	-96.11	23.97
>6 and <=8	-141.57	0.003	-235.44	-47.70
>8 and <=19	54.83	0.354	-61.14	170.81
Unknown	-16.52	0.429	-57.41	24.37
Training practice (No = reference)				
Yes	45.61	0.001	18.26	72.96
Unknown	-			
Nurse consultation rate (centred, per 1000 per 10,000 person years)	0.40	0.658	-1.36	2.15
GP consultation rate (centred, per 1000 per 10,000 person years)	-3.83	0.000	-4.80	-2.85
QOF performance (1st quintile = reference)				
2nd quintile	3.50	0.869	-38.00	44.99
3rd quintile	-16.22	0.427	-56.19	23.76
4th quintile	27.47	0.147	-9.67	64.61
5th quintile (best performance)	18.21	0.343	-19.40	55.83
Unknown	-50.43	0.633	-257.64	156.78
Rural practice (Urban = reference)	17.21	0.309	-15.98	50.39
Mean duration	417.27	0.000	367.50	467.04

For peer review only

Table S3: Final model for consultations with a GP including a term for GP registrar role (post-hoc sensitivity analysis)

	Change in duration (seconds)	p-value	95% confidence interval	
Female gender (Male = reference)	7.40	0.000	5.16	9.63
Ethnic group (White = reference)				
Asian	4.54	0.181	-2.11	11.19
Chinese	-7.76	0.523	-31.58	16.07
Black	-7.18	0.102	-15.79	1.43
Mixed/ Other	3.82	0.340	-4.03	11.68
Unknown	-12.32	0.000	-14.80	-9.85
Index of multiple deprivation (1st quintile = reference)				
2nd quintile	0.52	0.776	-3.06	4.10
3rd quintile	-3.37	0.090	-7.27	0.53
4th quintile	-4.88	0.021	-9.03	-0.74
5th quintile (most deprived)	-6.62	0.006	-11.30	-1.94
Unknown	-8.24	0.165	-19.87	3.39
Smoking status (Non-smoker = reference)				
Current smoker	-3.11	0.053	-6.26	0.04
Ex-smoker	-0.10	0.945	-3.09	2.88
Unknown	15.56	0.000	11.52	19.60
Age group (0-14 years = reference)				
15-24	52.35	0.000	46.82	57.88
25-44	81.88	0.000	76.97	86.79
45-64	92.15	0.000	87.14	97.15
65-74	71.73	0.000	66.21	77.26
75+	64.95	0.000	59.52	70.38
Telephone consultation (Face-to-face = reference)	-295.45	0.000	-298.37	-292.53
Training practice (No = reference)				
Yes	21.92	0.073	-2.07	45.90
Unknown	133.11	0.107	-28.55	294.77
GP registrar conducting appointment (No=reference)	245.04	0.000	241.49	248.59
GP consultation rate (centred, per 1000 per 10,000 person years)	-3.33	0.000	-4.24	-2.41
Mean duration	460.60	0.000	443.91	477.30

For peer review only

Table S4: Characteristics of practices according to mean duration of consultation with a nurse

	Practice mean consultation duration (minutes) (N=307)					
	<5	≥5 and <8	≥8 and <10	≥10 and <12	≥12 and <15	≥15
**Number of practices (%)**	**3 (1.0)**	**59 (19.2)**	**103 (33.6)**	**71 (23.1)**	**54 (17.6)**	**17 (5.5)**
Mean list size (SD)	8,189.0 (6,263.3)	9,936.8 (4,101.1)	10,029.6 (4,506.8)	10,945.5 (5,387.0)	8,591.0 (3,987.7)	7,286.6 (3,564.8)
Training practice						
Yes: N (%)	2 (66.7)	24 (40.7)	43 (41.8)	37 (52.1)	15 (27.8)	5 (29.4)
Rurality						
Not rural (Urban >10K - less sparse):N (%)	3 (100.0)	50 (84.8)	86 (83.5)	59 (83.1)	47 (87.0)	15 (88.2)
Mean GP consultation rate (per 10,000 person years) (SD)	34,558.4 (7,497.0)	39,556.1 (17,065.8)	39,333.7 (13,086.5)	37,073.1 (11,166.8)	34,315.5 (8,097.0)	35,907.5 (13,077.5)
Mean nurse consultation rate (per 10,000 person years) (SD)	15,359.3 (18,373.0)	17,422.4 (7,482.5)	15,404.9 (7,757.8)	12,162.1 (5,803.8)	9,868.6 (4,282.7)	7,029.6 (3,980.1)
Number of FTE GPs						
<=2: N (%)	1 (33.3)	5 (8.5)	10 (9.7)	6 (8.5)	12 (22.2)	5 (29.4)
>2 and <=4: N (%)	1 (33.3)	14 (23.7)	28 (27.2)	10 (14.1)	16 (29.6)	4 (23.5)
>4 and <=6: N (%)	0 (0.0)	22 (37.3)	27 (26.2)	31 (43.4)	13 (24.1)	6 (35.3)
>6 and <=8: N (%)	1 (33.3)	10 (17.0)	22 (21.4)	11 (15.5)	9 (16.7)	2 (11.8)
>8 and <=19: N (%)	0 (0.0)	7 (11.9)	16 (15.5)	13 (18.3)	4 (7.4)	0 (0.0)
Number of FTE nurses						
<=2: N (%)	2 (66.7)	35 (59.3)	51 (49.5)	42 (59.2)	38 (70.4)	12 (70.6)
>2 and <=4: N (%)	1 (33.3)	12 (20.3)	28 (27.2)	15 (21.2)	7 (13.0)	2 (11.8)
>4 and <=6: N (%)	0 (0.0)	3 (5.1)	11 (10.7)	5 (7.0)	1 (19.9)	0 (0.0)
>6 and <=8: N (%)	0 (0.0)	1 (1.7)	0 (0.0)	4 (5.6)	1 (1.9)	0 (0.0)
>8 and <=19: N (%)	0 (0.0)	1 (1.7)	2 (1.9)	1 (1.4)	0 (0.0)	0 (0.0)
QOF performance						
1st quintile (poorest performance): N (%)	1 (33.3)	9 (15.3)	15 (14.6)	13 (18.3)	7 (13.0)	2 (11.8)
2nd quintile: N (%)	0 (0.0)	5 (8.5)	17 (16.5)	13 (18.3)	10 (18.5)	1 (5.9)
3rd quintile: N (%)	0 (0.0)	9 (15.3)	24 (23.3)	13 (18.3)	7 (13.0)	6 (35.3)
4th quintile: N (%)	1 (33.3)	17 (28.8)	28 (27.2)	20 (28.2)	11 (20.4)	4 (23.5)
5th quintile (best performance): N (%)	1 (33.3)	17 (28.8)	19 (18.5)	12 (16.9)	19 (35.2)	4 (23.5)

For peer review only

Table S5: Full model for consultations with a nurse

	Change in duration (seconds)	p-value	95% confidence interval	
Female gender (Male = reference)	-11.15	0.000	-15.34	-6.96
Ethnic group (White = reference)				
Asian	0.18	0.979	-12.99	13.35
Chinese	-17.82	0.445	-63.57	27.93
Black	-1.67	0.849	-18.84	15.50
Mixed/ Other	-5.52	0.489	-21.18	10.13
Unknown	-2.52	0.279	-7.09	2.04
Index of multiple deprivation (1st quintile = reference)				
2nd quintile	7.49	0.026	0.92	14.06
3rd quintile	-0.54	0.883	-7.70	6.62
4th quintile	5.38	0.167	-2.24	13.01
5th quintile (most deprived)	7.92	0.071	-0.67	16.51
Unknown	-25.47	0.011	-45.10	-5.84
Smoking status (Non-smoker = reference)				
Current smoker	26.40	0.000	20.49	32.31
Ex-smoker	15.00	0.000	9.59	20.42
Unknown	21.12	0.000	13.23	29.01
Age group (0-14 years = reference)				
15-24	52.88	0.000	42.16	63.60
25-44	72.19	0.000	62.83	81.55
45-64	113.49	0.000	103.97	123.00
65-74	73.96	0.000	63.81	84.10
75+	75.54	0.000	65.43	85.65
Telephone consultation (Face-to-face = reference)	-279.51	0.000	-288.35	-270.66
Practice list size (centred, per 1000)	-6.09	0.065	-12.56	0.38
Number of FTE GPs (≤2 = reference)				
>2 and <=4	-63.99	0.045	-126.58	-1.39
>4 and <=6	-49.06	0.150	-115.82	17.70
>6 and <=8	-33.73	0.442	-119.68	52.22
>8 and <=19	-29.10	0.596	-136.61	78.42
Unknown	-106.02	0.625	-531.17	319.12
Number of FTE nurses (≤2 = reference)				
>2 and <=4	20.31	0.408	-27.79	68.41
>4 and <=6	49.99	0.240	-33.47	133.46
>6 and <=8	113.35	0.088	-16.77	243.48
>8 and <=19	137.25	0.096	-24.40	298.89
Unknown	-7.81	0.791	-65.58	49.97
Training practice (No = reference)				
Yes	-11.09	0.570	-49.31	27.14
Unknown	-			
Nurse consultation rate, centred, per 1000 per 10,000 person years)	-10.23	0.000	-12.84	-7.62
GP consultation rate, centred, per 1000 per 10,000 person years)	-0.49	0.488	-1.88	0.90
QOF performance (1st quintile = reference)				
2nd quintile	28.82	0.345	-30.93	88.56
3rd quintile	6.08	0.835	-50.99	63.14
4th quintile	9.11	0.738	-44.24	62.47
5th quintile (best performance)	31.22	0.254	-22.44	84.89
Unknown	-81.78	0.575	-367.98	204.42
Rural practice (Urban = reference)	13.93	0.561	-33.09	60.96
Mean duration	543.41	0.000	470.92	615.90

For peer review only

STROBE 2007 (v4) Statement—Checklist of items that should be included in reports of *cross-sectional studies*

Section/Topic	Item #	Recommendation	Reported on page #
**Title and abstract**	1	(*a*) Indicate the study's design with a commonly used term in the title or the abstract	1
		(*b*) Provide in the abstract an informative and balanced summary of what was done and what was found	2
**Introduction**			
Background/rationale	2	Explain the scientific background and rationale for the investigation being reported	3
Objectives	3	State specific objectives, including any prespecified hypotheses	3
**Methods**			
Study design	4	Present key elements of study design early in the paper	3
Setting	5	Describe the setting, locations, and relevant dates, including periods of recruitment, exposure, follow-up, and data collection	3
Participants	6	(*a*) Give the eligibility criteria, and the sources and methods of selection of participants	3
Variables	7	Clearly define all outcomes, exposures, predictors, potential confounders, and effect modifiers. Give diagnostic criteria, if applicable	4
Data sources/ measurement	8*	For each variable of interest, give sources of data and details of methods of assessment (measurement). Describe comparability of assessment methods if there is more than one group	3
Bias	9	Describe any efforts to address potential sources of bias	*3/4*
Study size	10	Explain how the study size was arrived at	3
Quantitative variables	11	Explain how quantitative variables were handled in the analyses. If applicable, describe which groupings were chosen and why	3
Statistical methods	12	(*a*) Describe all statistical methods, including those used to control for confounding	4
		(*b*) Describe any methods used to examine subgroups and interactions	N/A
		(*c*) Explain how missing data were addressed	4
		(*d*) If applicable, describe analytical methods taking account of sampling strategy	N/A
		(*e*) Describe any sensitivity analyses	7
**Results**			

For peer review only

Participants	13*	(a) Report numbers of individuals at each stage of study—eg numbers potentially eligible, examined for eligibility, confirmed eligible, included in the study, completing follow-up, and analysed	4
		(b) Give reasons for non-participation at each stage	4
		(c) Consider use of a flow diagram	N/A
Descriptive data	14*	(a) Give characteristics of study participants (eg demographic, clinical, social) and information on exposures and potential confounders	5/6
		(b) Indicate number of participants with missing data for each variable of interest	5/6
Outcome data	15*	Report numbers of outcome events or summary measures	5
Main results	16	(*a*) Give unadjusted estimates and, if applicable, confounder-adjusted estimates and their precision (eg, 95% confidence interval). Make clear which confounders were adjusted for and why they were included	7/8 and supplement
		(*b*) Report category boundaries when continuous variables were categorized	7/8
		(*c*) If relevant, consider translating estimates of relative risk into absolute risk for a meaningful time period	N/A
Other analyses	17	Report other analyses done—eg analyses of subgroups and interactions, and sensitivity analyses	7 and supplement
**Discussion**			
Key results	18	Summarise key results with reference to study objectives	9
Limitations	19	Discuss limitations of the study, taking into account sources of potential bias or imprecision. Discuss both direction and magnitude of any potential bias	9
Interpretation	20	Give a cautious overall interpretation of results considering objectives, limitations, multiplicity of analyses, results from similar studies, and other relevant evidence	10
Generalisability	21	Discuss the generalisability (external validity) of the study results	10
**Other information**			
Funding	22	Give the source of funding and the role of the funders for the present study and, if applicable, for the original study on which the present article is based	11

*Give information separately for cases and controls in case-control studies and, if applicable, for exposed and unexposed groups in cohort and cross-sectional studies.

Note: An Explanation and Elaboration article discusses each checklist item and gives methodological background and published examples of transparent reporting. The STROBE checklist is best used in conjunction with this article (freely available on the Web sites of PLoS Medicine at http://www.plosmedicine.org/, Annals of Internal Medicine at http://www.annals.org/, and Epidemiology at http://www.epidem.com/). Information on the STROBE Initiative is available at www.strobe-statement.org.

For peer review only

BMJ Open

Patient and practice level factors associated with consultation duration: a cross-sectional analysis of over 1 million consultations in English primary care.

Journal:	*BMJ Open*
Manuscript ID	bmjopen-2017-018261.R2
Article Type:	Research
Date Submitted by the Author:	10-Oct-2017
Complete List of Authors:	Stevens, Sarah; University of Oxford, Nuffield Department of Primary Care Health Sciences<br>Bankhead, Clare; University of Oxford<br>Mukhtar, Toqir; Nuffield Department of Primary Care Health Sciences<br>Perera, Rafael; University of Oxford, Primary Health Care<br>Holt, Tim; Oxford University, Department of Primary Care Health Sciences<br>Salisbury, Chris; University of Bristol, Academic Unit of Primary Health Care<br>Hobbs, Richard; University of Oxford, Dept of Primary Health Care
**Primary Subject Heading**:	General practice / Family practice
Secondary Subject Heading:	Health services research
Keywords:	PRIMARY CARE, Quality in health care < HEALTH SERVICES ADMINISTRATION & MANAGEMENT, Organisation of health services < HEALTH SERVICES ADMINISTRATION & MANAGEMENT, Health policy < HEALTH SERVICES ADMINISTRATION & MANAGEMENT

SCHOLARONE™
Manuscripts

For peer review only

Patient and practice level factors associated with consultation duration: a cross-sectional analysis of over 1 million consultations in English primary care.

Sarah Stevens[1], Clare Bankhead[1], Toqir Mukhtar[1], Rafael Perera-Salazar[1], Tim Holt[1], Chris Salisbury[2], FD Richard Hobbs[1], on behalf of the NIHR School for Primary Care Research, Nuffield Department of Primary Care Health Sciences, University of Oxford

1. Nuffield Department of Primary Care Health Sciences, University of Oxford, Oxford, UK
2. Centre for Academic Primary Care, School of Social and Community Medicine, University of Bristol, Bristol, UK.

Corresponding author:

Sarah Stevens,

Contact details: sarah.stevens@phc.ox.ac.uk, 01865289449

Address: Nuffield Department of Primary Care Health Sciences, Radcliffe Primary Care Building, Radcliffe Observatory Quarter, Woodstock Road, Oxford, OX2 6GG;

Word count: 2997

For peer review only - http://bmjopen.bmj.com/site/about/guidelines.xhtml

ABSTRACT

Objectives: Consultation duration has previously been shown to be associated with patient, practitioner, and practice characteristics. However, previous studies were conducted outside the UK, considered only small numbers of GP consultations, or focused primarily on practitioner level characteristics. We aimed to determine the patient and practice level factors associated with duration of GP and nurse consultations in UK primary care.

Design and setting: Cross sectional data were obtained from English general practices contributing to the Clinical Practice Research Datalink (CPRD) linked to data on patient deprivation and practice staffing, rurality, and Quality and Outcomes Framework (QOF) achievement.

Participants: 218,304 patients, from 316 English general practices, consulting from 1st April 2013 to 31st March 2014.

Analysis: Multilevel mixed effects models described the association between consultation duration and patient and practice-level factors (patient age, gender, smoking status, ethnic group, deprivation and practice rurality, number of full time equivalent GPs/nurses, list size, consultation rate, quintile of overall QOF achievement, and training status).

Results: Mean duration of face-to-face GP consultations was 9.24 minutes and 5.32 minutes for telephone consultations. Nurse face-to-face and telephone consultations lasted 9.70 and 5.73 minutes on average, respectively. Longer GP consultation duration was associated with female patient gender, practice training status and older patient age. Shorter duration was associated with higher deprivation and consultation rate. Longer nurse consultation duration was associated with male patient gender, older patient age and ever smoking; and shorter duration with higher consultation rate. Observed differences in duration were small (e.g. GP consultations with female patients compared to male patients were 8 seconds longer on average).

Conclusions: Small observed differences in consultation duration indicate that patients are treated similarly regardless of background. Increased consultation duration may be beneficial for older or comorbid patients, but the benefits and costs of increased consultation duration require further study.

ARTICLE SUMMARY

Strengths and limitations of this study

- This is a large-scale analysis of over 1 million consultations, using data known to be representative of the UK population.
- We have considered factors associated with the duration of both GP and nurse consultations allowing comparison between the two.
- Appointment duration may be recorded with some error, but average durations were consistent with 10-minute appointment slots.
- We were unable to examine how GP / nurse characteristics are associated with consultation duration and this requires further study.

For peer review only

INTRODUCTION

Patient-facing general practice workload in England has increased by 16% since 2007.[1] This reflects an increase in both the rate and duration of consultations. Consultation duration may be influenced by patient, practitioner, and practice level characteristics. At the practice level, previous studies have shown that shorter consultation duration is associated with greater practice list size[2] and workload.[3] The influence of practice rurality (rural compared to urban) is unclear with some studies indicating that rurality is negatively associated with consultation duration[2,4], and others demonstrating a positive association.[5] Relevant practitioner characteristics associated with longer consultations include female gender,[6] older age[3,5], but conversely, lesser experience.[6] Finally, longer consultations have been shown to be associated with patient characteristics, including female gender,[3–5,7] older age,[2–5,7] greater number of presenting problems,[3–5,7,8] and higher level of education[3] or socioeconomic status.[5]

However, many previous studies have been conducted in countries other than the UK and findings may not be generalizable to the National Health Service.[2–5] Studies within the UK provide limited up-to-date evidence having been conducted some time ago using data on a relatively small number of consultations,[7] or having focused on practitioner level characteristics alone.[6] Although a 2013 paper studied the association between practice, practitioner, and patient level characteristics and the number of presenting problems, demonstrating that the number of presenting problems is also associated with consultation duration, direct links between patient and practice characteristics and duration were not studied.[8] Finally, previous work has considered duration of GP consultations only, despite nurse consultations accounting for approximately one quarter of the overall UK primary care consultation rate in 2013/14.[1] Hence, we aimed to determine the patient and practice characteristics associated with increased duration of GP and nurse consultations in UK primary care in contemporary data.

METHODS

Consultation and patient data were obtained from the Clinical Practice Research Datalink (CPRD), a research database of anonymised patient records drawn from over 600 UK general practices.[9] English practices consenting to CPRD's data linkage scheme were included in the study if they contributed data covering any part of the study period (1st April 2013 to 31st March 2014), and were defined as "up-to-standard" (CPRD definition of continuous high quality data recording fit for use in research). All non-temporary patients registered at eligible practices for at least one day during the study period were included. Due to data volume, analysis was limited to a 10% simple random sample from each age-sex strata of eligible patents and those who consulted at least once during the study period.

CPRD data was linked to practice data on staffing,[10] rurality,[11] and Quality and Outcomes Framework (QOF) performance measures,[12] and patient Index of Multiple Deprivation (IMD). IMD data was supplied in quintiles by CPRD, who link patient postcodes to publically available IMD scores and group data into quintiles at the English national level. Staffing, rurality, and QOF data was downloaded from NHS digital (formerly the Health and Social Care Information Centre), and continuous variables were grouped prior to linkage with CPRD data. This was a requirement of the Independent Scientific Advisory Committee (ISAC) to CPRD, to limit the possibility of identifying individual CPRD practices. The approved protocol (number 15_120R) is available from the authors.

Consultations in CPRD represent occasions on which a patient's electronic health record is opened. We analysed consultations that were identified as face-to-face or telephone consultations based on

For peer review only - http://bmjopen.bmj.com/site/about/guidelines.xhtml

the variable “consultation type”, and those with a GP or nurse only, as indicated by the variable “staff role”. We excluded consultations where the patient record was opened purely for administrative purposes by GPs, nurses, or administrative staff (e.g. to record test results) and home visit consultations (since recorded duration may merely represent the time taken to record the consultation after it has ended).

Mean consultation duration across practices was examined using histograms. Practices were grouped according to their average consultation duration (<5, ≥5 and <8, ≥8 and <10, ≥10 and <12, ≥12 and <15 and ≥15 minutes) and differences in their characteristics described.

Multilevel mixed effects models were used to model the association between patient and practice characteristics and duration of GP or nurse consultations separately. Patient factors included as fixed effects were age, gender, smoking status (current, former, never), ethnic group, and quintile of IMD. Fixed effects practice level factors included were rurality, number of full time equivalent (FTE) GPs, number of FTE nurses, list size (centred), rate of GP consultation (centred), rate of nurse consultation (centred), quintile of overall QOF achievement, and practice training status (yes or no). Indicators for the patient and practice were included as random effects. All variables were entered into the models simultaneously and subsequently excluded in a stepwise fashion based on Z tests (binary and continuous variables), or chi-squared tests (categorical variables) at the 5% level. Missing smoking status and ethnic group data were included as separate categories in the models.

RESULTS

In total, 3,049,320 patients were eligible during the study period, of which 304,937 were randomly selected for inclusion. Of these, 218,304 consulting patients from 316 practices were included. The characteristics of the included patients and practices are given in Table 1 and Table 2. During the study period 964,148 consultations were conducted by a GP, and 347,657 were conducted by a nurse. The majority of consultations (1,155,040; 88%) were face-to-face consultations. Mean duration of face-to-face GP consultations was 9.24 (SD=8.06) minutes compared to 5.32 (6.21) minutes for telephone consultations. Nurse consultations were longer, on average, than those with GPs; face-to-face and telephone nurse consultations lasted 9.70 (9.21) and 5.73 (6.29) minutes. A minority of practices conducted substantially shorter or longer consultations on average (Figure S1).

For peer review only - http://bmjopen.bmj.com/site/about/guidelines.xhtml

Table 1: Characteristics of included patients (N=218,304)

	Mean/ N	SD/ %
Female gender	121,107	55.5
Age group		
0-14 years	36,371	16.7
15-24	23,020	10.5
25-44	55,316	25.3
45-64	57,000	26.1
65-74	24,086	11.0
75+	22,511	10.3
Smoking status		
Non-smoker	82,327	37.7
Current smoker	37,286	17.1
Ex-smoker	40,834	18.7
Unknown	57,857	26.5
Index of multiple deprivation		
1st quintile (least deprived)	48,363	22.2
2nd quintile	47,948	22.0
3rd quintile	41,825	19.2
4th quintile	41,953	19.2
5th quintile (most deprived)	34,750	15.9
Unknown	3,465	1.6
Ethnic group		
White	118,063	54.1
Asian	6,008	2.8
Chinese	491	0.2
Black	3,908	1.8
Mixed/ Other	4,374	2.0
Unknown	85,460	39.2

For peer review only

For peer review only - http://bmjopen.bmj.com/site/about/guidelines.xhtml

Table 2: Characteristics of included practices (N=316)

	Mean/ N	SD/ %
List size	9,649.7	4,648.4
Training practice		
Yes	126	39.9
Unknown	2	0.6
Rurality		
Not rural (Urban >10K - less sparse)	267	84.5
Rural (Hamlet/village/town & fringe)	49	15.5
GP consultation rate (per 10,000 person years)	37,441.0	13,043.6
Nurse consultation rate (per 10,000 person years)	13,217.3	7,580.5
Number of FTE GPs		
<=2	44	13.9
>2 and <=4	74	23.4
>4 and <=6	101	32.0
>6 and <=8	55	17.4
>8 and <=19	40	12.7
Unknown	2	0.6
Number of FTE nurses		
<=2	188	59.5
>2 and <=4	65	20.6
>4 and <=6	20	6.3
>6 and <=8	6	1.9
>8 and <=19	4	1.1
Unknown	33	10.4
QOF performance		
1st quintile (poorest performance)	50	15.8
2nd quintile	49	15.5
3rd quintile	59	18.7
4th quintile	82	26.0
5th quintile (best performance)	73	23.1
Unknown	3	1.0

GP consultations

Practice characteristics by average length of GP consultation, are described in Table S1. Practices conducting longer consultations had a lower rate of GP consultation but the relationship between other characteristics was less clear. Full model results for duration of GP consultations are given in Table S2 and variables were excluded in the following order: rate of nurse consultation (p=0.658), rurality (p=0.295), QOF performance (p=0.204), FTE nurses (p=0.063), FTE GPs (p=0.115), and list size (p=0.552). This yielded the final model in Table 3. Female patients' GP consultations were 8.3 seconds longer on average, and patients aged 0 to 14 years had the shortest consultations. Those aged 45-64 had the longest consultations; consultations were 1.5 minutes longer, on average, than consultations in 0 to 14 year olds. Although both ethnic group (p<0.001) and smoking status (p<0.001) were retained in the model, only the unknown categories showed significant associations: consultations with patients of unknown ethnicity were 11 seconds shorter than those with White patients, and consultations with patients of unknown smoking status were 19 seconds longer than those with non-smokers.

For peer review only - http://bmjopen.bmj.com/site/about/guidelines.xhtml

Table 3: Factors associated with duration of GP consultations

	Change in duration (seconds)	p-value	95% confidence interval	
Female gender (Male = reference)	8.29	0.000	6.03	10.55
Ethnic group (White = reference)				
Asian	4.06	0.237	-2.67	10.78
Chinese	-6.40	0.603	-30.49	17.69
Black	-5.70	0.200	-14.40	3.01
Mixed/ Other	4.30	0.289	-3.64	12.24
Unknown	-11.01	0.000	-13.51	-8.50
Index of multiple deprivation (1st quintile = reference)				
2nd quintile	1.14	0.537	-2.48	4.76
3rd quintile	-2.41	0.230	-6.35	1.53
4th quintile	-3.64	0.089	-7.83	0.56
5th quintile (most deprived)	-5.11	0.034	-9.84	-0.37
Unknown	-11.36	0.058	-23.12	0.39
Smoking status (Non-smoker = reference)				
Current smoker	-2.36	0.147	-5.54	0.83
Ex-smoker	0.11	0.943	-2.91	3.13
Unknown	18.65	0.000	14.56	22.73
Age group (0-14 years = reference)				
15-24	55.70	0.000	50.11	61.29
25-44	83.66	0.000	78.70	88.63
45-64	89.81	0.000	84.75	94.87
65-74	65.82	0.000	60.23	71.40
75+	58.43	0.000	52.94	63.92
Telephone consultation (Face-to-face = reference)	-308.71	0.000	-311.65	-305.77
Training practice (No = reference)				
Yes	44.33	0.000	19.87	68.78
Unknown	121.58	0.148	-43.02	286.17
GP consultation rate (centred, per 1000 per 10,000 person years)	-3.31	0.000	-4.24	-2.37
Mean duration	472.42	0.000	455.41	489.43

Duration of consultation decreased with increasing deprivation; consultations with patients in the most deprived quintile lasted 5 seconds less on average than consultations with the least deprived patients. Consultations in training practices were 44 seconds longer than those in practices that did not have trainee GPs, and telephone consultations were, on average, 5 minutes shorter than face-to-face consultations. Finally, for every 10% increase in consultation rate (1000 per 10,000 person years), GP consultation duration decreased by 3 seconds.

In post-hoc sensitivity analysis, we explored whether the association of duration with practice training status may be driven by consultations with trainee GPs alone, by adding a variable into the final model to indicate whether the GP conducting the consultation was a registrar or not. We found that consultations were on average 245 seconds longer with a GP registrar than otherwise and practice training status became non-significant (p=0.0656, Table S3).

Nurse consultations

Practice characteristics, by average length of nurse consultation, are described in Table S4. Similarly to GP consultations, practices conducting longer consultations had a lower rate of nurse

For peer review only - http://bmjopen.bmj.com/site/about/guidelines.xhtml

For peer review only

consultation. Full model results for duration of nurse consultations are given in Table S5. Variables were removed from the full model as follows: ethnic group (p=0.838), QOF performance (p=0.767), training practice (p=0.544), rurality (p=0.522), rate of GP consultation (p=0.547), FTE nurses (p=0.284), and list size (p=0.250).

In the final model (Table 4) consultations with a nurse were 11 seconds shorter for women than for men. All age groups had longer consultations than those aged 0 to 14 years (up to maximum of 2 minutes longer in those aged 45-64). Current smokers and ex-smokers had longer nurse consultations than non-smokers, by an average of 27 and 15 seconds, respectively. Those in the 2nd quintile of deprivation had longer consultations than those in the least deprived quintile, but there was no clear relationship in other groups. Those with unknown deprivation had shorter consultations. In practices with more than two FTE GPs, nurse consultations were between 78 and 86 seconds shorter, although the effect of FTE GPs was marginally significant (p=0.046), and was not significant when including list size in the model (p=0.109). Practices with a higher rate of nurse consultation had shorter consultations by an average of 9 seconds for every 10% increase in consultation rate (1000 consultations per 10,000 person years).

Table 4: Factors associated with duration of nurse consultations

	Change in duration (seconds)	p-value	95% confidence interval	
Female gender (Male = reference)	-11.06	0.000	-15.24	-6.88
Index of multiple deprivation (1st quintile = reference)				
2nd quintile	7.58	0.024	1.01	14.16
3rd quintile	-0.40	0.912	-7.56	6.75
4th quintile	5.49	0.158	-2.14	13.11
5th quintile (most deprived)	8.04	0.066	-0.53	16.62
Unknown	-26.93	0.007	-46.39	-7.46
Smoking status (Non-smoker = reference)				
Current smoker	26.67	0.000	20.80	32.55
Ex-smoker	15.20	0.000	9.80	20.60
Unknown	21.06	0.000	13.17	28.94
Age group (0-14 years = reference)				
15-24	52.30	0.000	41.70	62.91
25-44	71.85	0.000	62.54	81.16
45-64	113.15	0.000	103.70	122.59
65-74	73.81	0.000	63.71	83.90
75+	75.68	0.000	65.61	85.75
Telephone consultation (Face-to-face = reference)	-279.34	0.000	-288.18	-270.50
Number of FTE GPs (≤2 = reference)				
>2 and <=4	-85.82	0.004	-144.44	-27.20
>4 and <=6	-82.57	0.004	-138.53	-26.61
>6 and <=8	-82.90	0.008	-144.25	-21.55
>8 and <=19	-78.14	0.020	-143.96	-12.32
Unknown	-235.93	0.140	-549.15	77.29
Nurse consultation rate, centred, per 1000 per 10,000 person years)	-9.19	0.000	-11.53	-6.84
Mean duration	598.72	0.000	549.66	647.78

For peer review only - http://bmjopen.bmj.com/site/about/guidelines.xhtml

DISCUSSION

We have shown that duration of consultation is associated with both patient and practice level characteristics. Increasing patient age is associated with increased consultation duration. Female patient gender increases the length of GP consultations and decreases the length of nurse consultations and duration of nurse consultations is increased in current and ex-smokers. GP consultations are longer in practices involved in GP training and with less deprived patients, but shorter in practices with a higher consultation rate. Although there is some variation in mean duration across practices, this is not explained by many of the practice characteristics studied.

Strengths and limitations

This is a large-scale analysis of over 1 million consultations across England, and therefore provides reliable estimates of association. Moreover, CPRD is broadly representative of the UK population,[9] and our results are likely to be representative of those consulting across England. A further strength is our separate consideration of GP and nurse consultations, allowing us to describe factors associated with the length of nurse consultations for the first time. A limitation is the consideration of general practice consultations only and our results may not be generalizable to other settings (e.g. walk in centres).

Consultation duration in CPRD reflects the length of time a patient record is open within the practice computer system, recorded in whole minutes. There were instances in the data of very long (>60 minutes, 0.3%) and very short (apparent 0 minutes, 8.3%) consultations which we rounded to 60 minutes and 0.5 minutes respectively. Long consultations may occur for genuine clinical need, but also if a staff member forgets to close a record. Apparent short consultations may occur if a record is opened incorrectly, if details of a straightforward consultation are entered only at the end of a consultation, or if the type of consultation (e.g. administrative) was miscoded. However, average durations were in line with a standard 10-minute appointment window, and final model estimates were similar when excluding these extreme durations or including them without rounding (data not shown).

Due to missing data, we included some “Unknown” categories in our models. Previous research has shown that former smoking is under-reported in CPRD compared to UK national survey data,[13] so those with unknown status in this study may be more likely to be former smokers. Ethnic group data was drawn from hospital episodes data, so those with missing data may be healthier and consult less often (ethnic group was missing in 39% of patients, but only in 29% of consultations). Hence consultations in these patients may have been shorter and less complex. Ethnicity data is similarly poorly reported in CPRD,[9] hence, more detailed data is required to fully explore these associations.

We did not have data on GP and nurse characteristics, so were unable to examine their association with duration. Previous research outside of the UK has shown that consultations with older GPs are longer,[3,5] but UK-based research indicates that consultations are longer in those with lesser experience.[6] Our results regarding the association of duration with practice training status and GP registrar status are consistent with the UK research. However, we found that in practices which were not identified as training practices in the national data, 4.9% of GP consultations appeared to be conducted by GP registrars (compared to 11.6% in training practices). This indicates inaccuracies in coding either of staff role or of training practice status, and hence this finding needs further replication in future studies.

We did not examine the relationship between consultation duration and the number of presenting problems. A 2010 study indicated that GP consultation duration may be increased by 2 minutes for

For peer review only - http://bmjopen.bmj.com/site/about/guidelines.xhtml

For peer review only

each additional presenting problem.[8] A similar large-scale analysis using CPRD presents many methodological difficulties and is the subject of ongoing work by the study authors.

Comparison with the literature

Our contemporary results confirm previous research findings that increasing duration of GP consultation is associated with older patient age, [2–5,7] female patient gender,[3–7] and socioeconomic status.[5] Older patient age and current or prior smoking are also associated with increased duration of nurse consultations. Although female patients have longer GP consultations, they have shorter nurse consultations, and the reason for this is unclear. Nurses may conduct more relatively straightforward consultations with women (e.g. contraception reviews) compared to with men.

Consultations are shorter in practices with a greater corresponding consultation rate, perhaps indicating that appointment lengths are limited to meet consultation demand, with little spare capacity in schedules. This is consistent with our previous work[1] showing that GP workload has increased by 16% in England since 2007.[1] Conversely, more problems may be dealt with in a longer consultation, reducing the need for repeat consults. This was previously demonstrated by a study in two practices where increased initial consultation duration was associated with a lower consultation rate in the following 4 weeks.[14]

Implications

We observed small absolute differences in consultation duration, despite statistical significance for some factors. This may suggest that all patients are treated similarly and that consultation duration is equitable and in line with patient need. For example we observed large differences related to patient age, which is likely to be confounded with comorbidity and complexity of consultation. However, our findings that more deprived patients have shorter consultations on average could indicate inequalities based on clinical need since more deprived patients have higher rates of premature mortality.[15] Practices with an older or comorbid patient list could increase the length of scheduled appointments to better match the required consultation time in these patients. Practices could also allow patients to choose their consultation length. This has been shown to improve doctor and patient experience, and patients could be educated to estimate their required time.[16]

Patients in the 31.7% of practices offering consultations less than eight minutes long may receive significantly less GP care compared to those in the 31% of practices providing consultations of 10 or more minutes long, particularly when considering this difference across multiple appointments. However, we observed a small decrease in duration of three seconds for every 10% increase in consultation rate indicating a degree of trade-off between consultation length and number. The importance of consultation duration partly depends on its association with outcomes. Increasing duration has been shown to increase patient enablement, and decrease GP stress.[17] A previous review suggested that doctors conducting longer consultations are more likely to offer health promotion advice, and deal with long-term problems.[18] Longer consultations may also reduce prescribing rates,[18] and be associated with more appropriate prescribing.[19] However there is little strong evidence that duration is associated with patient satisfaction generally,[18,20] or when GPs are pre-selected for poor communication,[21] although it may be associated with more patient centeredness.[6]

Evidence from exploratory trials suggests that increasing consultation duration (as part of a wider complex intervention) is highly cost-effective.[22] However, a recent review indicated that many studies assessing interventions to alter consultation duration are at high risk of bias; the effect of

For peer review only - http://bmjopen.bmj.com/site/about/guidelines.xhtml

altering duration on the number of referrals, prescriptions, or patient satisfaction is uncertain.[23] Further research is required to establish the benefits and costs of increasing consultation duration alone.

GP consultations are longer on average in practices hosting trainees. This may have implications for the future of general practice since GP recruitment has not kept pace with growth in the consulting population, and fewer trainees intend to stay in full time clinical work.[24] Policy makers and those responsible for recruitment should consider how the increased time required to train GPs can be accommodated given increasing workload pressures.[1]

COMPETING INTERESTS

All authors have completed the ICMJE uniform disclosure form at www.icmje.org/coi_disclosure.pdf and declare: SS, CS and RP report grants from the National Institute for Health Research School for Primary Care Research, during the conduct of the study; no financial relationships with any organisations that might have an interest in the submitted work in the previous three years; no other relationships or activities that could appear to have influenced the submitted work.

FUNDING

This project is funded by the National Institute for Health Research School for Primary Care Research (NIHR SPCR). FDRH is partly supported as a NIHR Senior Investigator, Director of the NIHR SPCR, Director of the NIHR CLARHC Oxford, Theme Leader of the NIHR Oxford BRC, NIHR Oxford DEC, and as a Professorial Fellow of Harris Manchester College. The views expressed are those of the author(s) and not necessarily those of the NHS, the NIHR or the Department of Health.

AUTHOR CONTRIBUTIONS

FDRH and CS conceived the research, obtained funding, and are joint principal investigators. FDRH, CB, and CS drafted the protocol, which SS, TM, RP and TH all then contributed to. SS and TM were responsible for data management, and SS did the statistical analyses. SS drafted the report, which FDRH, CS, CB, RP, TM, and TH then contributed to. SS is the guarantor and corresponding author.

DATA SHARING

Data is available from CPRD directly: www.cprd.com.

For peer review only

For peer review only - http://bmjopen.bmj.com/site/about/guidelines.xhtml

REFERENCES

1 Hobbs FDR, Bankhead C, Mukhtar T, *et al.* Clinical workload in UK primary care: a retrospective analysis of 100 million consultations in England, 2007–14. *Lancet* 2016;**387**:2323–30. doi:10.1016/S0140-6736(16)00620-6

2 van den Berg MJ, de Bakker DH, Wester GP, *et al.* Do list size and remuneration affect GPs' decisions about how they provide consultations? *BMC Health Serv Res* 2009;**9**. doi:10.1016/0277-9536(91)90087-S

3 Petek-Ster M, Svab I, Zivcec GK. Factors related to consultation time: experience in Slovenia. *Scand J Prim Health Care* 2008;**26**:29–34. doi:10.1080/02813430701760789

4 Deveugele M, Derese A, Brink-muinen A Van Den, *et al.* Consultation Length In General Practice: Cross Sectional Study. *BMJ* 2002;**325**:472–4.

5 Britt HC, Valenti L, Miller GC. Determinants of consultation length in Australian general practice. *Med J Aust* 2005;**183**:68–71.

6 Orton PK, Pereira Gray D. Factors influencing consultation length in general/family practice. *Fam Pract* 2016;**33**:529–34. doi:10.1093/fampra/cmw056

7 Carr-Hill R, Jenkins-Clarke S, Dixon P, *et al.* Do minutes count? Consultation lengths in general practice. *J Health Serv Res Policy* 1998;**3**:207–13.

8 Salisbury C, Procter S, Stewart K, *et al.* The content of general practice consultations: Cross-sectional study based on video recordings. *Br J Gen Pract* 2013;**63**:e751–9. doi:10.3399/bjgp13X674431

9 Herrett E, Gallagher AM, Bhaskaran K, *et al.* Data Resource Profile: Clinical Practice Research Datalink (CPRD). *Int J Epidemiol* 2015;**44**:827–36. doi:10.1093/ije/dyv098

10 Health and Social Care Information Centre. General and personal medical services: England, 2004–2014, As at 30 September. 2015.http://content.digital.nhs.uk/catalogue/PUB16934 (accessed 11 May2015).

11 NHS Digital. Dataset: Rural/Urban definition of GP practice: categorical, 2011. 2011.https://indicators.hscic.gov.uk/webview/ (accessed 11 May2015).

12 Health and Social Care Information Centre. Quality and Outcomes Framework (QOF) - 2013-14. 2014.http://content.digital.nhs.uk/catalogue/PUB15751 (accessed 11 May2015).

13 Booth HP, Prevost AT, Gulliford MC. Validity of smoking prevalence estimates from primary care electronic health records compared with national population survey data for England, 2007 to 2011. *Pharmacoepidemiol Drug Saf* 2013;**22**:1357–61. doi:10.1002/pds.3537

14 Hughes D. Consultation length and outcome in two group general practices. *J R Coll Gen Pract* 1983;**33**:143–7.

15 Kontopantelis E, Springate DA, Ashworth M, *et al.* Investigating the relationship between quality of primary care and premature mortality in England: a spatial whole-population study. *BMJ* 2015;**350**:h904.

16 Sampson R, O'Rourke J, Hendry R, *et al.* Sharing control of appointment length with patients in general practice. *Br J Gen Pract* 2013;**63**:e185–91. doi:10.3399/bjgp13X668096

17 Mercer SW, Fitzpatrick B, Gourlay G, *et al.* More time for complex consultations in a high-deprivation practice is associated with increased patient enablement. *Br J Gen Pract* 2007;**57**:960–6. doi:10.3399/096016407782604910

For peer review only - http://bmjopen.bmj.com/site/about/guidelines.xhtml

18 Wilson A, Childs S. The effect of interventions to alter the consultation length of family physicians: A systematic review. *Br J Gen Pract* 2006;**56**:876–82.

19 Heaney DJ, Walker JJ, Howie JGR, *et al.* The development of a routine NHS data-based index of performance in general practice (NHSPPI). *Fam Pract* 2002;**19**:77–84.

20 Lemon TI, Smith RH. Consultation Content not Consultation Length Improves Patient Satisfaction. *J Fam Med Prim care* 2014;**3**:333–9. doi:10.4103/2249-4863.148102

21 Elmore N, Burt J, Abel G, *et al.* Investigating the relationship between consultation length and patient experience: a cross-sectional study in primary care. *Br J Gen Pract* 2016;**66**.

22 Mercer SW, Fitzpatrick B, Guthrie B, *et al.* The CARE Plus study – a whole-system intervention to improve quality of life of primary care patients with multimorbidity in areas of high socioeconomic deprivation: exploratory cluster randomised controlled trial and cost-utility analysis. *BMC Med* 2016;**14**. doi:10.1136/bmj.h176

23 Wilson AD, Childs S, Gonçalves-Bradley DC, *et al.* Interventions to increase or decrease the length of primary care physicians' consultation. *Cochrane database Syst Rev* 2016;**8**:CD003540. doi:10.1002/14651858.CD003540.pub3

24 Baird B, Charles A, Honeyman M, *et al. Understanding pressures in general practice*. The Kings Fund 2016.

For peer review only

For peer review only - http://bmjopen.bmj.com/site/about/guidelines.xhtml

Supplement

Figure S1: Distribution of mean consultation duration in each practice for face-to-face or telephone consultations conducted by a GP or nurse

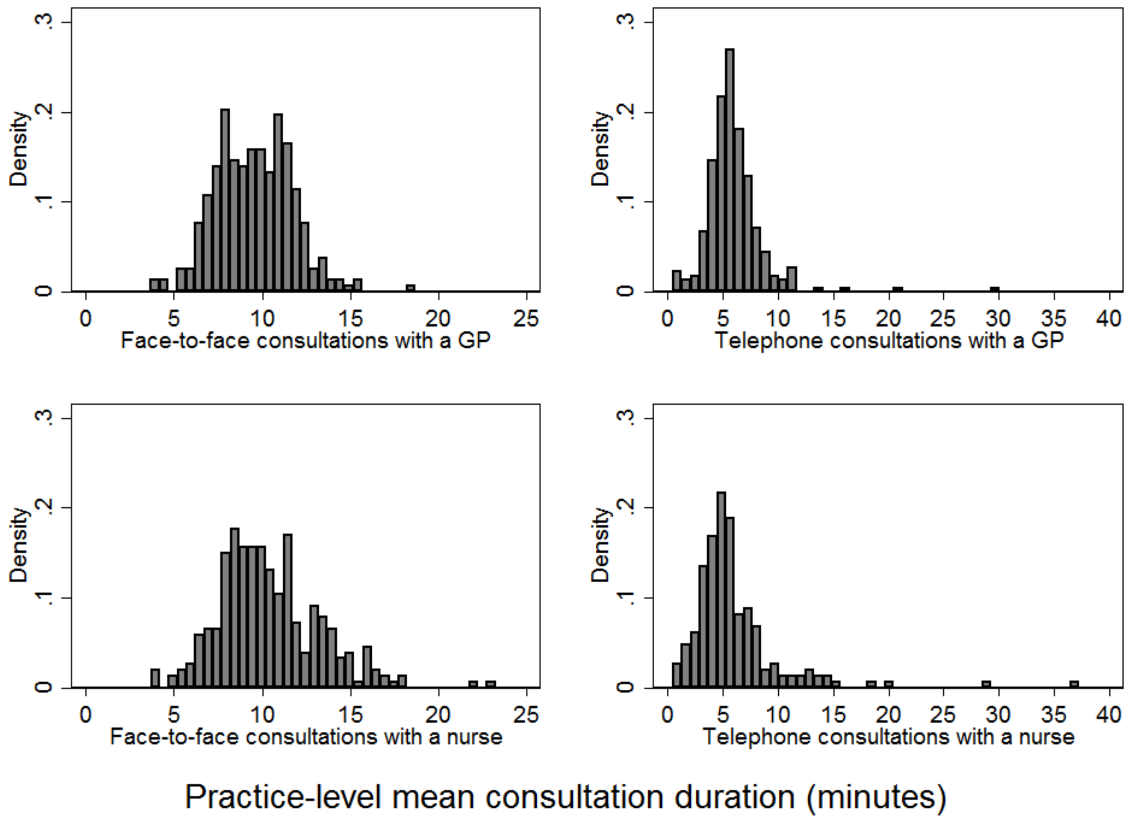

Table S1: Characteristics of practices according to mean duration of consultation with a GP

	Practice mean consultation duration (minutes) (N=316)					
	**<5**	**≥5 and <8**	**≥8 and <10**	**≥10 and <12**	**≥12 and <15**	**≥15**
**Number of practices (%)**	**4 (1.3)**	**96 (30.4)**	**118 (37.3)**	**83 (26.3)**	**14 (4.4)**	**1 (0.3)**
Mean list size (SD)	5,580.0 (713.0)	10,149.3 (4,603.6)	10,232.1 (4809.5)	8,771.6 (4,590.9)	7,715.2 (2,912.3)	9,220.0 (-)
Training practice						
Yes: N (%)	0 (0.0)	33 (34.4)	53 (44.9)	35 (42.2)	5 (35.7)	0 (0.0)
Rurality						
Not rural (Urban >10K - less sparse):N (%)	4 (100.0)	82 (85.4)	101 (85.6)	68 (81.9)	12 (85.7)	0 (0.0)
Mean GP consultation rate (per 10,000 person years) (SD)	46,877.5 (17,478.6)	42,488.2 (16,711.8)	37,671.8 (9,954.9)	32,618.1 (9,227.8)	28,463.9 (9,387.5)	13,895.5 (-)
Mean nurse consultation rate (per 10,000 person years) (SD)	12,777.7 (4,657.8)	14,270.9 (7,893.9)	13,566.1 (7,677.1)	11,707.4 (6,888.7)	12,279.2 (8,778.4)	11,124.7 (-)
Number of FTE GPs						
<=2: N (%)	2 (50.0)	9 (9.4)	16 (13.6)	14 (16.9)	3 (21.4)	0 (0.0)
>2 and <=4: N (%)	2 (50.0)	24 (25.0)	22 (18.6)	24 (28.9)	2 (14.3)	0 (0.0)
>4 and <=6: N (%)	0 (0.0)	37 (38.5)	32 (27.1)	24 (28.9)	7 (50.0)	1 (100.0)
>6 and <=8: N (%)	0 (0.0)	15 (15.6)	25 (21.2)	15 (18.1)	0 (0.0)	0 (0.0)
>8 and <=19: N (%)	0 (0.0)	11 (11.5)	22 (18.6)	5 (6.0)	2(14.3)	0 (0.0)
Number of FTE nurses						
<=2: N (%)	4 (100.0)	51 (53.1)	69 (58.5)	52 (62.7)	12 (85.7)	0 (0.0)
>2 and <=4: N (%)	0 (0.0)	22 (22.9)	22 (18.6)	18 (21.7)	2 (14.3)	1 (100.0)
>4 and <=6: N (%)	0 (0.0)	8 (8.3)	10 (8.5)	2 (2.4)	0 (0.0)	0 (0.0)
>6 and <=8: N (%)	0 (0.0)	4 (4.2)	2 (1.7)	0 (0.0)	0 (0.0)	0 (0.0)
>8 and <=19: N (%)	0 (0.0)	2 (2.1)	2 (1.7)	0 (0.0)	0 (0.0)	0 (0.0)
QOF performance						
1st quintile (poorest performance): N (%)	2 (50.0)	15 (15.6)	18 (15.3)	12 (14.5)	3 (21.4)	0 (0.0)
2nd quintile: N (%)	0 (0.0)	22(22.9)	13 (11.0)	13 (15.7)	1 (7.1)	0 (0.0)
3rd quintile: N (%)	1 (25.0)	23 (24.0)	24 (20.3)	8 (9.6)	2 (14.3)	1 (100.0)
4th quintile: N (%)	1 (25.0)	18 (18.8)	31 (26.3)	27 (32.5)	5 (35.7)	0 (0.0)
5th quintile (best performance): N (%)	0 (0.0)	18 (18.8)	30 (25.4)	22 (26.5)	3 (21.4)	0 (0.0)

For peer review only

Table S2: Full model for consultations with a GP

	Change in duration (seconds)	p-value	95% confidence interval	
Female gender (Male = reference)	8.29	0.000	6.03	10.55
Ethnic group (White = reference)				
Asian	4.11	0.231	-2.61	10.84
Chinese	-6.32	0.607	-30.41	17.76
Black	-5.63	0.205	-14.34	3.07
Mixed/ Other	4.35	0.283	-3.59	12.29
Unknown	-11.00	0.000	-13.50	-8.49
Index of multiple deprivation (1st quintile = reference)				
2nd quintile	1.14	0.535	-2.47	4.76
3rd quintile	-2.39	0.235	-6.33	1.55
4th quintile	-3.57	0.095	-7.77	0.62
5th quintile (most deprived)	-5.04	0.037	-9.77	-0.30
Unknown	-11.27	0.060	-23.02	0.48
Smoking status (Non-smoker = reference)				
Current smoker	-2.36	0.147	-5.54	0.83
Ex-smoker	0.11	0.944	-2.91	3.13
Unknown	18.66	0.000	14.58	22.75
Age group (0-14 years = reference)				
15-24	55.71	0.000	50.12	61.30
25-44	83.69	0.000	78.72	88.65
45-64	89.83	0.000	84.77	94.89
65-74	65.84	0.000	60.25	71.42
75+	58.44	0.000	52.96	63.93
Telephone consultation (Face-to-face = reference)	-308.70	0.000	-311.64	-305.76
Practice list size (centred, per 1000)	-5.21	0.026	-9.81	-0.61
Number of FTE GPs (≤2 = reference)				
>2 and <=4	27.74	0.206	-15.23	70.70
>4 and <=6	56.90	0.016	10.62	103.18
>6 and <=8	58.06	0.061	-2.61	118.73
>8 and <=19	120.02	0.002	43.58	196.47
Unknown	211.75	0.120	-54.93	478.44
Number of FTE nurses (≤2 = reference)				
>2 and <=4	-10.07	0.568	-44.59	24.46
>4 and <=6	-36.07	0.239	-96.11	23.97
>6 and <=8	-141.57	0.003	-235.44	-47.70
>8 and <=19	54.83	0.354	-61.14	170.81
Unknown	-16.52	0.429	-57.41	24.37
Training practice (No = reference)				
Yes	45.61	0.001	18.26	72.96
Unknown	-			
Nurse consultation rate (centred, per 1000 per 10,000 person years)	0.40	0.658	-1.36	2.15
GP consultation rate (centred, per 1000 per 10,000 person years)	-3.83	0.000	-4.80	-2.85
QOF performance (1st quintile = reference)				
2nd quintile	3.50	0.869	-38.00	44.99
3rd quintile	-16.22	0.427	-56.19	23.76
4th quintile	27.47	0.147	-9.67	64.61
5th quintile (best performance)	18.21	0.343	-19.40	55.83
Unknown	-50.43	0.633	-257.64	156.78
Rural practice (Urban = reference)	17.21	0.309	-15.98	50.39
Mean duration	417.27	0.000	367.50	467.04

Table S3: Final model for consultations with a GP including a term for GP registrar role (post-hoc sensitivity analysis)

	Change in duration (seconds)	p-value	95% confidence interval	
Female gender (Male = reference)	7.40	0.000	5.16	9.63
Ethnic group (White = reference)				
Asian	4.54	0.181	-2.11	11.19
Chinese	-7.76	0.523	-31.58	16.07
Black	-7.18	0.102	-15.79	1.43
Mixed/ Other	3.82	0.340	-4.03	11.68
Unknown	-12.32	0.000	-14.80	-9.85
Index of multiple deprivation (1st quintile = reference)				
2nd quintile	0.52	0.776	-3.06	4.10
3rd quintile	-3.37	0.090	-7.27	0.53
4th quintile	-4.88	0.021	-9.03	-0.74
5th quintile (most deprived)	-6.62	0.006	-11.30	-1.94
Unknown	-8.24	0.165	-19.87	3.39
Smoking status (Non-smoker = reference)				
Current smoker	-3.11	0.053	-6.26	0.04
Ex-smoker	-0.10	0.945	-3.09	2.88
Unknown	15.56	0.000	11.52	19.60
Age group (0-14 years = reference)				
15-24	52.35	0.000	46.82	57.88
25-44	81.88	0.000	76.97	86.79
45-64	92.15	0.000	87.14	97.15
65-74	71.73	0.000	66.21	77.26
75+	64.95	0.000	59.52	70.38
Telephone consultation (Face-to-face = reference)	-295.45	0.000	-298.37	-292.53
Training practice (No = reference)				
Yes	21.92	0.073	-2.07	45.90
Unknown	133.11	0.107	-28.55	294.77
GP registrar conducting appointment (No=reference)	245.04	0.000	241.49	248.59
GP consultation rate (centred, per 1000 per 10,000 person years)	-3.33	0.000	-4.24	-2.41
Mean duration	460.60	0.000	443.91	477.30

For peer review only

Table S4: Characteristics of practices according to mean duration of consultation with a nurse

	Practice mean consultation duration (minutes) (N=307)					
	**<5**	**≥5 and <8**	**≥8 and <10**	**≥10 and <12**	**≥12 and <15**	**≥15**
**Number of practices (%)**	**3 (1.0)**	**59 (19.2)**	**103 (33.6)**	**71 (23.1)**	**54 (17.6)**	**17 (5.5)**
Mean list size (SD)	8,189.0 (6,263.3)	9,936.8 (4,101.1)	10,029.6 (4,506.8)	10,945.5 (5,387.0)	8,591.0 (3,987.7)	7,286.6 (3,564.8)
Training practice						
Yes: N (%)	2 (66.7)	24 (40.7)	43 (41.8)	37 (52.1)	15 (27.8)	5 (29.4)
Rurality						
Not rural (Urban >10K - less sparse):N (%)	3 (100.0)	50 (84.8)	86 (83.5)	59 (83.1)	47 (87.0)	15 (88.2)
Mean GP consultation rate (per 10,000 person years) (SD)	34,558.4 (7,497.0)	39,556.1 (17,065.8)	39,333.7 (13,086.5)	37,073.1 (11,166.8)	34,315.5 (8,097.0)	35,907.5 (13,077.5)
Mean nurse consultation rate (per 10,000 person years) (SD)	15,359.3 (18,373.0)	17,422.4 (7,482.5)	15,404.9 (7,757.8)	12,162.1 (5,803.8)	9,868.6 (4,282.7)	7,029.6 (3,980.1)
Number of FTE GPs						
<=2: N (%)	1 (33.3)	5 (8.5)	10 (9.7)	6 (8.5)	12 (22.2)	5 (29.4)
>2 and <=4: N (%)	1 (33.3)	14 (23.7)	28 (27.2)	10 (14.1)	16 (29.6)	4 (23.5)
>4 and <=6: N (%)	0 (0.0)	22 (37.3)	27 (26.2)	31 (43.4)	13 (24.1)	6 (35.3)
>6 and <=8: N (%)	1 (33.3)	10 (17.0)	22 (21.4)	11 (15.5)	9 (16.7)	2 (11.8)
>8 and <=19: N (%)	0 (0.0)	7 (11.9)	16 (15.5)	13 (18.3)	4 (7.4)	0 (0.0)
Number of FTE nurses						
<=2: N (%)	2 (66.7)	35 (59.3)	51 (49.5)	42 (59.2)	38 (70.4)	12 (70.6)
>2 and <=4: N (%)	1 (33.3)	12 (20.3)	28 (27.2)	15 (21.2)	7 (13.0)	2 (11.8)
>4 and <=6: N (%)	0 (0.0)	3 (5.1)	11 (10.7)	5 (7.0)	1 (19.9)	0 (0.0)
>6 and <=8: N (%)	0 (0.0)	1 (1.7)	0 (0.0)	4 (5.6)	1 (1.9)	0 (0.0)
>8 and <=19: N (%)	0 (0.0)	1 (1.7)	2 (1.9)	1 (1.4)	0 (0.0)	0 (0.0)
QOF performance						
1st quintile (poorest performance): N (%)	1 (33.3)	9 (15.3)	15 (14.6)	13 (18.3)	7 (13.0)	2 (11.8)
2nd quintile: N (%)	0 (0.0)	5 (8.5)	17 (16.5)	13 (18.3)	10 (18.5)	1 (5.9)
3rd quintile: N (%)	0 (0.0)	9 (15.3)	24 (23.3)	13 (18.3)	7 (13.0)	6 (35.3)
4th quintile: N (%)	1 (33.3)	17 (28.8)	28 (27.2)	20 (28.2)	11 (20.4)	4 (23.5)
5th quintile (best performance): N (%)	1 (33.3)	17 (28.8)	19 (18.5)	12 (16.9)	19 (35.2)	4 (23.5)

For peer review only

Table S5: Full model for consultations with a nurse

	Change in duration (seconds)	p-value	95% confidence interval	
Female gender (Male = reference)	-11.15	0.000	-15.34	-6.96
Ethnic group (White = reference)				
Asian	0.18	0.979	-12.99	13.35
Chinese	-17.82	0.445	-63.57	27.93
Black	-1.67	0.849	-18.84	15.50
Mixed/ Other	-5.52	0.489	-21.18	10.13
Unknown	-2.52	0.279	-7.09	2.04
Index of multiple deprivation (1st quintile = reference)				
2nd quintile	7.49	0.026	0.92	14.06
3rd quintile	-0.54	0.883	-7.70	6.62
4th quintile	5.38	0.167	-2.24	13.01
5th quintile (most deprived)	7.92	0.071	-0.67	16.51
Unknown	-25.47	0.011	-45.10	-5.84
Smoking status (Non-smoker = reference)				
Current smoker	26.40	0.000	20.49	32.31
Ex-smoker	15.00	0.000	9.59	20.42
Unknown	21.12	0.000	13.23	29.01
Age group (0-14 years = reference)				
15-24	52.88	0.000	42.16	63.60
25-44	72.19	0.000	62.83	81.55
45-64	113.49	0.000	103.97	123.00
65-74	73.96	0.000	63.81	84.10
75+	75.54	0.000	65.43	85.65
Telephone consultation (Face-to-face = reference)	-279.51	0.000	-288.35	-270.66
Practice list size (centred, per 1000)	-6.09	0.065	-12.56	0.38
Number of FTE GPs (≤2 = reference)				
>2 and <=4	-63.99	0.045	-126.58	-1.39
>4 and <=6	-49.06	0.150	-115.82	17.70
>6 and <=8	-33.73	0.442	-119.68	52.22
>8 and <=19	-29.10	0.596	-136.61	78.42
Unknown	-106.02	0.625	-531.17	319.12
Number of FTE nurses (≤2 = reference)				
>2 and <=4	20.31	0.408	-27.79	68.41
>4 and <=6	49.99	0.240	-33.47	133.46
>6 and <=8	113.35	0.088	-16.77	243.48
>8 and <=19	137.25	0.096	-24.40	298.89
Unknown	-7.81	0.791	-65.58	49.97
Training practice (No = reference)				
Yes	-11.09	0.570	-49.31	27.14
Unknown	-			
Nurse consultation rate, centred, per 1000 per 10,000 person years)	-10.23	0.000	-12.84	-7.62
GP consultation rate, centred, per 1000 per 10,000 person years)	-0.49	0.488	-1.88	0.90
QOF performance (1st quintile = reference)				
2nd quintile	28.82	0.345	-30.93	88.56
3rd quintile	6.08	0.835	-50.99	63.14
4th quintile	9.11	0.738	-44.24	62.47
5th quintile (best performance)	31.22	0.254	-22.44	84.89
Unknown	-81.78	0.575	-367.98	204.42
Rural practice (Urban = reference)	13.93	0.561	-33.09	60.96
Mean duration	543.41	0.000	470.92	615.90

For peer review only

STROBE 2007 (v4) Statement—Checklist of items that should be included in reports of *cross-sectional studies*

Section/Topic	Item #	Recommendation	Reported on page #
**Title and abstract**	1	(*a*) Indicate the study's design with a commonly used term in the title or the abstract	1
		(*b*) Provide in the abstract an informative and balanced summary of what was done and what was found	2
**Introduction**			
Background/rationale	2	Explain the scientific background and rationale for the investigation being reported	3
Objectives	3	State specific objectives, including any prespecified hypotheses	3
**Methods**			
Study design	4	Present key elements of study design early in the paper	3
Setting	5	Describe the setting, locations, and relevant dates, including periods of recruitment, exposure, follow-up, and data collection	3
Participants	6	(*a*) Give the eligibility criteria, and the sources and methods of selection of participants	3
Variables	7	Clearly define all outcomes, exposures, predictors, potential confounders, and effect modifiers. Give diagnostic criteria, if applicable	4
Data sources/ measurement	8*	For each variable of interest, give sources of data and details of methods of assessment (measurement). Describe comparability of assessment methods if there is more than one group	3
Bias	9	Describe any efforts to address potential sources of bias	*3/4*
Study size	10	Explain how the study size was arrived at	3
Quantitative variables	11	Explain how quantitative variables were handled in the analyses. If applicable, describe which groupings were chosen and why	3
Statistical methods	12	(*a*) Describe all statistical methods, including those used to control for confounding	4
		(*b*) Describe any methods used to examine subgroups and interactions	N/A
		(*c*) Explain how missing data were addressed	4
		(*d*) If applicable, describe analytical methods taking account of sampling strategy	N/A
		(*e*) Describe any sensitivity analyses	7
**Results**			

For peer review only

Participants	13*	(a) Report numbers of individuals at each stage of study—eg numbers potentially eligible, examined for eligibility, confirmed eligible, included in the study, completing follow-up, and analysed	4
		(b) Give reasons for non-participation at each stage	4
		(c) Consider use of a flow diagram	N/A
Descriptive data	14*	(a) Give characteristics of study participants (eg demographic, clinical, social) and information on exposures and potential confounders	5/6
		(b) Indicate number of participants with missing data for each variable of interest	5/6
Outcome data	15*	Report numbers of outcome events or summary measures	5
Main results	16	(*a*) Give unadjusted estimates and, if applicable, confounder-adjusted estimates and their precision (eg, 95% confidence interval). Make clear which confounders were adjusted for and why they were included	7/8 and supplement
		(*b*) Report category boundaries when continuous variables were categorized	7/8
		(*c*) If relevant, consider translating estimates of relative risk into absolute risk for a meaningful time period	N/A
Other analyses	17	Report other analyses done—eg analyses of subgroups and interactions, and sensitivity analyses	7 and supplement
**Discussion**			
Key results	18	Summarise key results with reference to study objectives	9
Limitations	19	Discuss limitations of the study, taking into account sources of potential bias or imprecision. Discuss both direction and magnitude of any potential bias	9
Interpretation	20	Give a cautious overall interpretation of results considering objectives, limitations, multiplicity of analyses, results from similar studies, and other relevant evidence	10
Generalisability	21	Discuss the generalisability (external validity) of the study results	10
**Other information**			
Funding	22	Give the source of funding and the role of the funders for the present study and, if applicable, for the original study on which the present article is based	11

*Give information separately for cases and controls in case-control studies and, if applicable, for exposed and unexposed groups in cohort and cross-sectional studies.

Note: An Explanation and Elaboration article discusses each checklist item and gives methodological background and published examples of transparent reporting. The STROBE checklist is best used in conjunction with this article (freely available on the Web sites of PLoS Medicine at http://www.plosmedicine.org/, Annals of Internal Medicine at http://www.annals.org/, and Epidemiology at http://www.epidem.com/). Information on the STROBE Initiative is available at www.strobe-statement.org.

For peer review only
